# Chemoselective synthesis of diaryl disulfides via a visible light-mediated coupling of arenediazonium tetrafluoroborates and CS_2_

**DOI:** 10.3762/bjoc.13.91

**Published:** 2017-05-15

**Authors:** Jing Leng, Shi-Meng Wang, Hua-Li Qin

**Affiliations:** 1 School of Chemistry, Chemical Engineering and Life Science, Wuhan University of Technology, 205 Luoshi Road, Wuhan 430070, PR China

**Keywords:** arenediazonium tetrafluoroborates, carbon disulfide, chemoselectivity, diaryl disulfides, photocatalyst

## Abstract

A highly efficient and chemoselective method for the synthesis of diaryl disulfides is developed via a visible light-promoted coupling of readily accessible arenediazonium tetrafluoroborates and CS_2_. This practical and convenient protocol provides a direct pathway for the assembly of a series of disulfides in an environmentally friendly manner with good to excellent yields.

## Findings

**Scheme 1:**
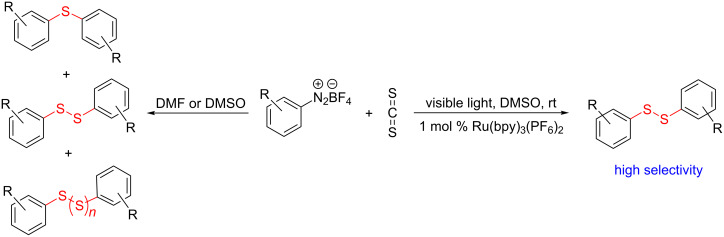
Chemoselective assembly of diaryl disulfides.

The development of methods for the functionalization of peptides and proteins under mild conditions is a current frontier in the fields of chemistry, biology and drug discovery [1–4]. Most of the pharmaceutically relevant proteins contain disulfide bonds, furthermore, the disulfide ligation and its established chemoselectivity is of great advantage for proteins’ functionalization [5]. In addition, disulfides also play valuable roles as versatile building blocks for industrial applications [6–8]. Thus, the development of methodologies for the synthesis of disulfides is rather desirable and many research groups have made great contributions to the synthesis of diaryl disulfides such as the Chandrasekaran group [9] and the Wacharasindhu group [10]. Indeed, the design of sustainable and useful transformations with applications in industry is considered of high practical value. In this context, carbon disulfide, a cheap and abundant chemical, has been widely used as reactant and solvent in both industry and materials science. For example, Batanero and co-workers reported an electrochemical transformation of carbon disulfide into diaryl disulfides [11]. Sunlight as abundant and almost infinitely available energy resource has been widely used for chemical transformations in the sense of cost, safety, availability, and environmental friendliness [12–15]. Herein, we report a visible light-mediated coupling of arenediazonium tetrafluoroborates and CS_2_ for the chemoselective assembly of diaryl disulfides as our continuing endeavor of utilizing arenediazonium tetrafluoroborates [16] for synthetic applications (Scheme 1).

We conducted our initial study with benzenediazonium tetrafluoroborate (**1a**) and CS_2_ (**2**) as model substrates to examine the feasibility of the formation of diphenyl disulfide (**3a**) (Table 1). Various solvents were screened and to our delight, it was found that the reaction of **1a** and **2** in DMF and DMSO gave the desired product in a moderate yield of 54% and 53%, respectively (Table 1, entries 7 and 8). Unfortunately, under the applied conditions, the chemoselectivity of the reaction was poor, affording a mixture of unexpected diphenyl sulfide (**4a**) and diphenyl polysulfides (**5a**) as byproducts. Thus, a study to optimize the reaction conditions with regard to chemoselectivity and to minimize the formation of the byproducts was conducted.

**Table 1:** Solvent screening for the coupling of benzenediazonium tetrafluoroborate (**1a**) and CS_2_ (**2**).^a^

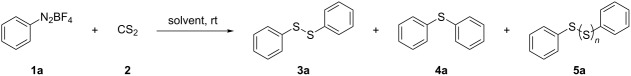				

Entry	Solvents	Yield **3a** (%)^b^	Yield **4a** (%)^b^	Yield **5a** (%)^b^

1	MeOH	n.d.	n.d.	n.d.
2	THF	36	5	n.d.
3	dioxane	n.d.	14	n.d.
4	acetone	n.d.	n.d.	n.d.
5	DCM	n.d.	n.d.	19
6	acetonitrile	n.d.	n.d.	n.d.
7	DMF	54	3	31
8	DMSO	53	3	27
9	hexane	n.d.	n.d.	24

^a^Reaction conditions: **1a** (0.1 mmol), **2** (0.2 mmol), solvent (2 mL), rt, 6 h; ^b^yields were determined by HPLC using **3a** and **4a** as the external standards; the yield of **5a** is based on the integration of the corresponding HPLC peaks [17–18]; n.d. = not determined.

As recently surveyed, photoredox catalysts are widely employed for the generation of radicals for diverse radical reactions [19]. Further, the application of aryl radicals generated from aryldiazonium salts under visible light irradiation has also been studied [14–15] by taking advantage of visible light as abundant and environmentally friendly energy source for organic syntheses. The photochemistry of diazonium salts has been widely studied since the early 19th century, at which time, it was noticed that benzenediazonium nitrate turns red upon exposure to sunlight due to decomposition and formation of radical species [20]. Subsequently, the photodecomposition of diazonium salts by loss of nitrogen upon exposure to light has been utilized in organic synthesis for example to remove amino groups from anilines [21] or for arylation reactions [15,22].

Based on the above research results, we envisioned that a radical pathway may facilitate the formation of diaryl disulfides. Therefore the photocatalyst Ru(bpy)_3_(PF_6_)_2_ (bpy = 2,2’-bipyridine) [23] and a 20 W blue-light LED were chosen as catalyst and the source of visible light, respectively for our model reaction (Table 2). A variety of solvents was evaluated and eventually, it was found that the coupling of benzenediazonium tetrafluoroborate (**1a**) and CS_2_ (**2**) in ethanol as the solvent gave the desired product diphenyl disulfide (**3a**) in 77% yield accompanied by only 8% of the undesired diphenyl polysulfides (Table 2, entry 4). Switching to DMSO as the solvent for the reaction afforded exclusively the desired product **3a** in excellent yield (88%, Table 2, entry 6). Next, other sulfur sources were also examined, such as S_8_, NaSH, Na_2_S, Na_2_S_2_O_3_, Na_2_S_2_O_4_ and K_2_S_2_O_8_, however, none of them provided the desired product in an acceptable yield (Table 2, entries 7–13).

**Table 2:** Screening of the solvents and sulfur sources for the visible light-mediated coupling of benzenediazonium tetrafluoroborate (**1a**) and CS_2_ (**2**) and other sulfur sources in the presence of Ru(bpy)_3_(PF_6_)_2_ as the photocatalyst.^a^

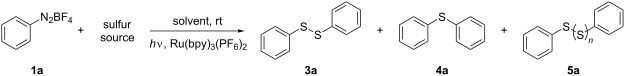					

Entry	Solvent	Sulfur source	Yield **3a** (%)^b^	Yield **4a** (%)^b^	Yield **5a** (%)^b^

1	MeOH	CS_2_	88	<1	10
2	H_2_O	CS_2_	47	5	20
3	THF	CS_2_	87	<1	8
4	EtOH	CS_2_	77	n.d.	8
5	acetone	CS_2_	79	7	3
6	DMSO	CS_2_	88	n.d.	<1
7	DMSO	S_8_	14	9	67
8	DMSO	Na_2_S	n.d.	43	11
9	DMSO	Na_2_S_2_O_3_	n.d.	n.d.	n.d.
10	DMSO	Na_2_S_2_O_4_	n.d.	n.d.	n.d.
11	DMSO	K_2_S_2_O_8_	n.d.	n.d.	4
12	DMSO	NaSH	22	28	14
13	DMSO	(NH_4_)_2_S_2_O_8_	n.d.	n.d.	4

^a^Reaction conditions: **1a** (0.1 mmol), sulfur sources (0.2 mmol), Ru(bpy)_3_(PF_6_)_2_ (0.001 mmol), blue light (20 W), solvents (2 mL), rt, 6 h; ^b^yields were determined by HPLC using **3a** and **4a** as the external standards, the yield of **5a** is based on the integration of the corresponding HPLC peaks [17–18]; n.d. = not determined.

In order to maximize the yields, varying amounts of CS_2_ (**2**) were also tested (Table 3) and it was found that the CS_2_ loading had a considerable influence on the reaction. By decreasing the loading of CS_2_ from 2 equiv to 0.5 equiv, the yield of the product **3a** dropped to 42%, whereas increasing amounts of CS_2_ did not significantly increase the yield of the product. Subsequently, different photocatalysts were investigated and it turned out that the choice of catalyst also had a significant impact on our model reaction. Ru(bpy)_3_Cl_2_ catalyzed this coupling to afford the desired product **3a** in a moderate yield of 65% (Table 3, entry 8). However, when the iridium-based photocatalysts Ir(ppy)_3_ [24], [Ir(ppy)_2_(bpy)]PF_6_ and [Ir(ppy)_2_(dtbbpy)]PF_6_ (bpy = 2,2’-bipyridine, ppy = 2-phenylpyridine, dtbbpy = 4,4’-di-*tert*-butyl-2,2’-bipyridine) [25–26] were used, the product yield of diphenyl disulfide (**3a**) was much lower compared to reactions performed in the presence of ruthenium catalysts (Table 3, entries 9–11).

**Table 3:** Screening of photocatalysts for the visible light-mediated coupling of benzenediazonium tetrafluoroborate (**1a**) and CS_2_ (**2**).^a^

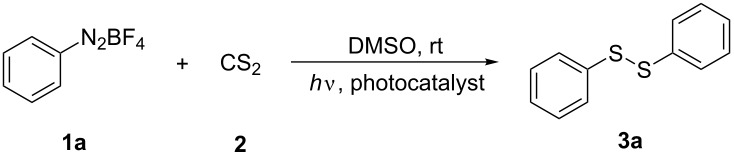				

Entry	**2** (equiv)	photocatalyst	solvent	Yield **3a** (%)^b^

1	0.5	Ru(bpy)_3_(PF_6_)_2_	DMSO	42
2	1	Ru(bpy)_3_(PF_6_)_2_	DMSO	47
3	1.5	Ru(bpy)_3_(PF_6_)_2_	DMSO	53
4	2	Ru(bpy)_3_(PF_6_)_2_	DMSO	88
5	2.5	Ru(bpy)_3_(PF_6_)_2_	DMSO	55
6	3	Ru(bpy)_3_(PF_6_)_2_	DMSO	57
7	–	Ru(bpy)_3_(PF_6_)_2_	CS_2_	n.d.
8	2	Ru(bpy)_3_Cl_2_	DMSO	65
9	2	Ir(ppy)_3_	DMSO	57
10	2	Ir(ppy)_2_(bpy)(PF_6_)	DMSO	73
11	2	Ir(ppy)_2_(dtbbpy)(PF_6_)	DMSO	8
12	2	none	DMSO	53

^a^Reaction conditions: **1a** (0.1 mmol), photocatalyst (0.001 mmol), blue light (20 W), solvent (2 mL), rt, 6 h; ^b^yields were determined by HPLC using **3a** as the external standard.

**Scheme 2:**
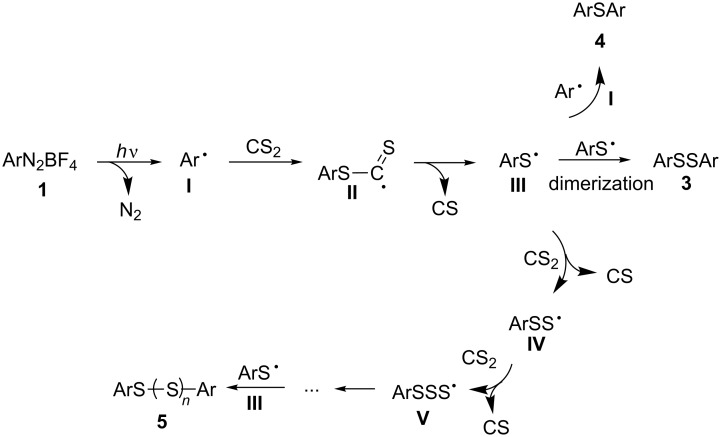
A plausible reaction mechanism.

A plausible reaction mechanism has been proposed and is depicted in Scheme 2. We envision that the phenyl radical **I** was initially generated under visible light irradiation [14–15]. Subsequently, the radical **I** attacked the sulfur atom of carbon disulfide to provide the intermediate **II** which can be converted to radical intermediate **III** through the cleavage of the carbon–sulfur bond accompanied with the release of a carbon sulfide [11]. The active radical intermediate **III** can transform into three types of products through different pathways. Firstly, diaryl disulfide **3** is obtained through a dimerization of radical intermediates **III**, whereas the reaction of radical **III** with phenyl radical **I** is leading to byproduct **4**. Finally, radical **III** can react with various equivalents of CS_2_ with release of carbon sulfide to generate aryl-polythio radicals **IV** and **V**. The combination of the latter intermediates with radical **I** then finally affords polysulfides **5**.

To demonstrate the scope of the reaction, a series of arenediazonium tetrafluoroborates was utilized in the reaction with CS_2_ to generate the corresponding diaryl disulfides (Table 4). Arenediazonium tetrafluoroborates **1b–p** with both, electron-withdrawing and donating groups successfully underwent transformation, affording the corresponding coupling products **3b–p** in good to excellent yields (42–99%). Also sterically demanding substrates gave the desired products in good yields (**3d**, **3f**, **3g**, **3i**, **3m** and **3n**) and functional groups such as chloro, bromo, ester, methyl, nitro, and phenyl groups were also compatible with the reaction conditions.

**Table 4:** Reaction scope of the visible light-mediated coupling of arenediazonium tetrafluoroborates **1** with CS_2_ (**2**).

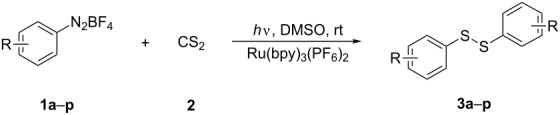			

Substrate **1**^a^	Product **3**, yield^b^	Substrate **1**^a^	Product **3**, yield^b^

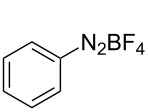 **1a**	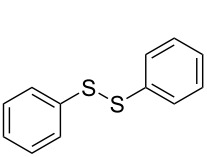 **3a**, 80%, 50%^c^	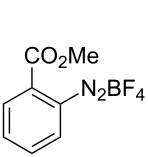 **1i**	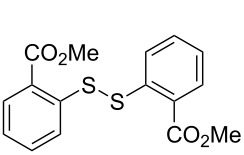 **3i**, 94%, 82%^c^
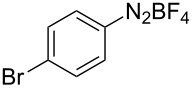 **1b**	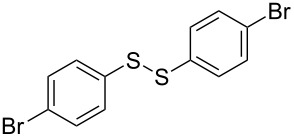 **3b**, 81%, 78%^c^	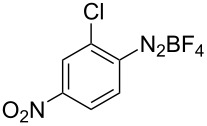 **1j**	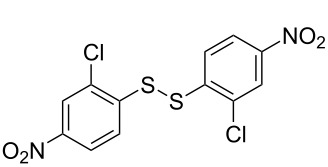 **3j**, 99%, 85%^c^
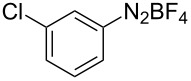 **1c**	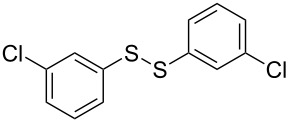 **3c**, 85%, 72%^c^	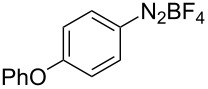 **1k**	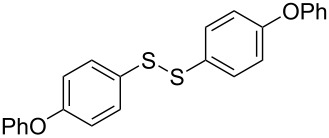 **3k**, 70%
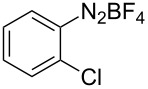 **1d**	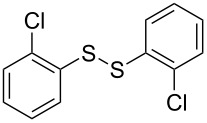 **3d**, 94%	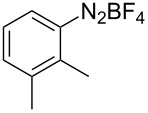 **1l**	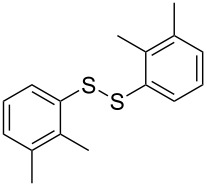 **3l**, 76%
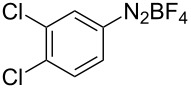 **1e**	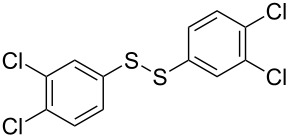 **3e**, 90%	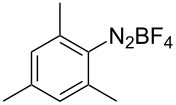 **1m**	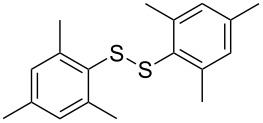 **3m**, 56%
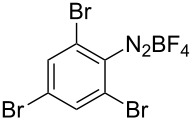 **1f**	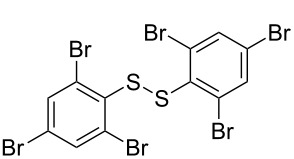 **3f**, 88%	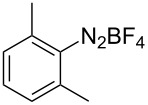 **1n**	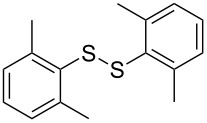 **3n**, 42%
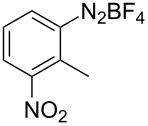 **1g**	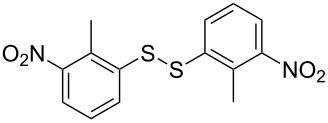 **3g**, 88%	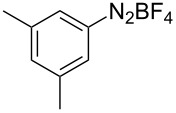 **1o**	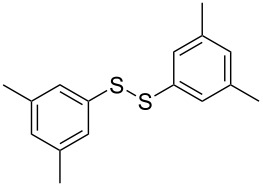 **3o**, 56%
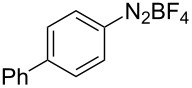 **1h**	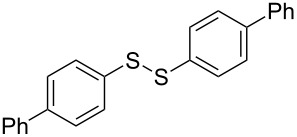 **3h**, 76%	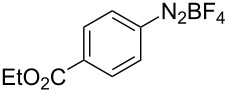 **1p**	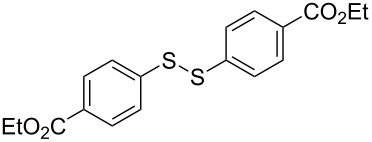 **3p**, 90%^c^, 92%^c,d^

^a^Reaction conditions: **1** (0.1 mmol), CS_2_ (0.2 mmol), Ru(bpy)_3_(PF_6_)_2_ (0.001 mmol), blue light (20 W), DMSO (2 mL), rt, 6 h; ^b^isolated yields after chromatography on silica gel; ^c^the reactions were carried out with the diazonium salts **1** at a 5 mmol scale; ^d^acetone was used as the solvent.

## Conclusion

In conclusion, we have developed an efficient method for the synthesis of diaryl disulfides through the coupling of arenediazonium tetrafluoroborates and CS_2_. This straightforward visible light-promoted process proceeds under mild reaction conditions and is applicable for the assembly of a wide range of diaryl disulfides. Further studies to clearly understand the reaction mechanism and the synthetic applications are ongoing in our laboratory.

## Supporting Information

File 1 Experimental procedures, characterization data and copies of ^1^H and ^13^C NMR spectra for final compounds.
