# Supplementary material for: Chemoselective synthesis of diaryl disulfides via a visible light-mediated coupling of arenediazonium tetrafluoroborates and CS2
Source: Beilstein J Org Chem. 2017 May 15;13:903–9. doi: 10.3762/bjoc.13.91 (PMC5480343; doi:10.3762/bjoc.13.91)
Supplement: File 1 — Experimental procedures, characterization data and copies of 1H and 13C NMR spectra for final compounds. [file Beilstein_J_Org_Chem-13-903-s001.pdf]

Supporting Information

for

Chemoselective synthesis of diaryl disulfides via a visible light-mediated coupling of arenediazonium tetrafluoroborates and CS_2

Jing Leng, Shi-Meng Wang and Hua-Li Qin*

Address: School of Chemistry, Chemical Engineering and Life Science, Wuhan University of Technology, 205 Luoshi Road, Wuhan 430070, PR China

Email: Hua-Li Qin*- qinhuali@whut.edu.cn

*Corresponding author

Experimental procedures, characterization data and copies of ^{1}H and ^{13}C NMR spectra for final compounds

Table of contents

1. General
2. General procedure for the synthesis of arenediazonium tetrafluoroborates
3. General procedure for the preparation of compounds **3a–p**
4. NMR spectra

1. General

All reactions were carried out in oven or flame-dried glassware under air atmosphere. Unless otherwise noted, solvents and reagents were used as purchased without further purification. The reactions were magnetically stirred and monitored by thin layer chromatography. Yields refer to chromatographically and spectroscopically pure compounds. ^{1}H and ^{13}C NMR spectra were recorded in $CDCl_3$ at 500 MHz (^{1}H NMR) and 126 MHz (^{13}C NMR) on a Bruker Avance 500 spectrometer. Chemical shifts are reported in parts per million relative to TMS using the residual solvent signal as internal standard ($CDCl_3$,7.26 and 77.2 ppm) and coupling constants are given in hertz (Hz). Data are reported as follows: chemical shift, multiplicity (s = singlet, d = doublet, t = triplet, q = quartet, m = multiplet, br = broad), coupling constant.

2. General procedure for the synthesis of arenediazonium tetrafluoroborates [1]

In a 250 mL round-bottomed flask, aniline (20.0 mmol) was dissolved in acetone (5 mL) and cooled to −20 °C. A 50% aqueous solution of HBF_4 (20 mL) was added. The mixture was kept at −20 °C with a bath of 70% water in methanol and dry ice before a solution of $NaNO_2$ (2.75 g, 40 mmol, dissolved in 5 mL H_2O) was added drop-wise. The reaction mixture was stirred at 0 °C (ice–water bath) for additional 20 min before cooled again to –20 °C for the arenediazonium tetrafluoroborate to precipitate (in some case, the salts precipitated at lower temperature). The crude diazonium salt was collected by filtration, washed successively with a small amount of ice water, methanol, and diethyl ether before dried in vacuo (3–10 mbar) for 30 min. Pure arenediazonium tetrafluoroborates were obtained by recrystallization from acetone.

3. General procedure for the preparation of compounds 3a–p

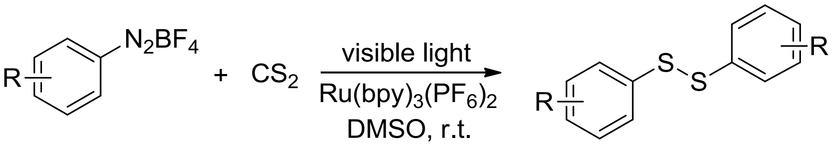

To a flask charged with the corresponding arenediazonium tetrafluoroborate (0.5 mmol) and carbon disulfide (1 mmol, 76 mg), DMSO (5 mL) and the photocatalyst $Ru(bpy)_3(PF_6)_2$ (1 mol %) were added at room temperature. The mixture was placed in the irradiation apparatus equipped with a 20 W blue light-emitting diode (LED) strip and the reaction progress was followed by TLC. After complete conversion of the starting material (approximately 5–6 h), the reaction mixture was extracted with EtOAc (3 × 15 mL). The combined organic phase was washed with H_2O (3 ×30 mL) to completely remove DMSO, dried ($MgSO_4$) and concentrated in vacuo. The products were purified by column chromatography on silica gel using petroleum ether or a mixture of petroleum ether and ethyl acetate as eluents.

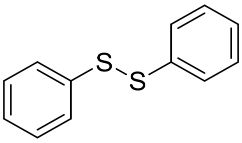

1,2-Diphenyldisulfane (**3a**) [2]

80%; white solid. ^{1}H NMR (500 MHz, $CDCl_3$): δ 7.50 (d, *J* = 8.0 Hz, 4H), 7.30 (t, *J* = 7.5 Hz, 4H), 7.23 (t, *J* =7.5 Hz, 2H); ^{13}C NMR (126 MHz, $CDCl_3$): δ 137.2, 129.2, 127.7, 127.3 .

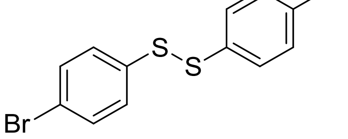

1,2-Bis(4-bromophenyl)disulfane (**3b**) [2]

81%; white solid. ^{1}H NMR (500 M, $CDCl_3$): δ 7.43 (d, *J* =8.6 Hz, 4H), 7.34 (d, *J* = 8.6 Hz, 4H); ^{13}C NMR (126 MHz, $CDCl_3$): δ 135.9, 132.4, 129.6, 121.7.

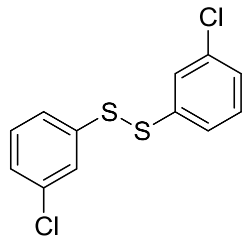

1,2-Bis(3-chlorophenyl)disulfane (**3c**) [3]

85%; white solid. ^{1}H NMR (500 MHz, $CDCl_3$): δ 7.48 (s, 2H), 7.36 (dt, J_1 = 7.4 Hz, J_2 = 1.8 Hz, 2H), 7.26-7.20 (m, 4H); ^{13}C NMR (126 MHz, $CDCl_3$): δ 138.5, 135.3, 130.3, 127.7, 127.1, 125.5.

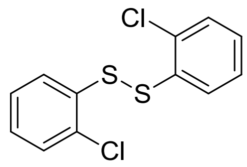

1,2-Bis(2-chlorophenyl)disulfane (**3d**) [4]

94%; white solid. ^{1}H NMR (500 MHz, $CDCl_3$): δ 7.56 (dd, J_1 = 7.9 Hz, J_2= 1.5 Hz, 2H), 7.37 (dd, J_1 =7.8 Hz, J_2 =1.3 Hz, 2H), 7.22 (td, J_1 = 7.7 Hz, J_2 = 1.2 Hz, 2H), 7.16 (td, J_1 = 7.6 Hz, J_2 =1.6 Hz, 2H); ^{13}C NMR (126 MHz, $CDCl_3$): δ 134.6, 132.0, 129.9, 128.0, 127.7, 127.4.

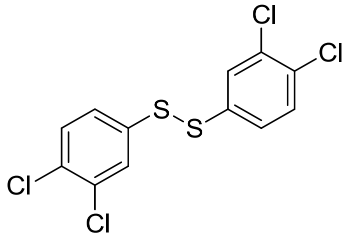

1,2-Bis(3,4-dichlorophenyl)disulfane (**3e**) [5]

90%; white solid. ^{1}H NMR (500 MHz, $CDCl_3$): δ 7.56 (d, J = 2.0 Hz, 2H), 7.38 (d, J = 8.4 Hz, 2H), 7.29 (dd, J_1 = 8.4 Hz, J_2 = 2.0 Hz, 2H); ^{13}C NMR (126 MHz, $CDCl_3$): δ 136.4, 133.7, 132.2, 131.2, 129.4, 127.1.

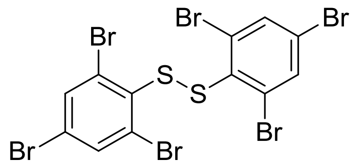

1,2-Bis(2,4,6-tribromophenyl)disulfane (**3f**) [6]

88%; white solid. ^{1}H NMR (500 MHz, $CDCl_3$): δ 7.74 (s, 4H); ^{13}C NMR (126 MHz, $CDCl_3$): δ 136.6, 135.4; 132.3; 125.0.

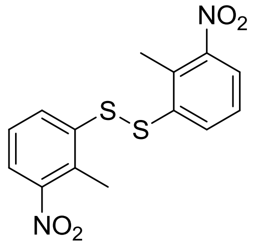

1,2-Bis(2-methyl-3-nitrophenyl)disulfane (**3g**) [7]

88%; white solid. ^{1}H NMR (500 MHz, $CDCl_3$): δ 7.71 (dd, J_1 = 9.5 Hz, J_2 = 1.9 Hz, 4H), 7.29 (t, J = 8.0 Hz, 2H), 2.59 (s, 6H); ^{13}C NMR (126 MHz, $CDCl_3$): δ 151.4, 138.3, 132.1, 131.5, 127.2, 123.5, 16.2.

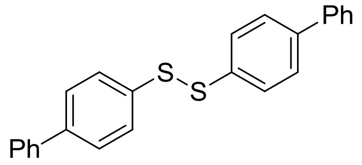

1,2-Di([1,1'-biphenyl]-4-yl)disulfane (**3h**) [8]

76%; white solid. ^{1}H NMR (500 MHz, $CDCl_3$): δ 7.62 (d, J = 8.5 Hz, 4H), 7.57(t, J = 7.5 Hz, 8H), 7.45 (t, J = 7.6 Hz, 4H), 7.36 (t, J =7.4 Hz, 2H); ^{13}C NMR (126 MHz, $CDCl_3$): δ 140.5, 140.3, 136.2, 129.0, 128.4, 127.9, 127.7, 127.1.

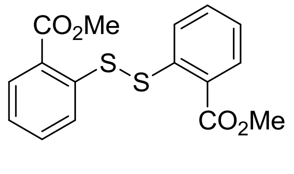

Dimethyl 2,2'-disulfanediyldibenzoate (**3i**) [9]

94%; white solid. ^{1}H NMR (500 MHz, $CDCl_3$): δ 8.06 (d, J = 7.8 Hz, 2H), 7.75 (d, J =

8.2 Hz, 2H), δ 7.40 (t, J = 7.7 Hz, 2H), δ 7.23 (t, J = 7.0 Hz, 2H), 3.98 (s, 6H); ^{13}C NMR (126 MHz, $CDCl_3$): δ 167.0, 140.5, 133.2, 131.6, 127.4, 126.0, 125.6, 52.5.

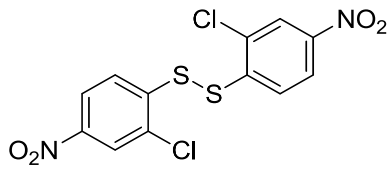

1,2-Bis(2-chloro-4-nitrophenyl)disulfane (**3j**) [10]

99%; white solid. ^{1}H NMR (500 MHz, $CDCl_3$): δ 8.29 (d, J = 2.3 Hz, 2H), 8.10 (dd, J_1 = 8.8 Hz, J_2 = 2.3 Hz, 2H), 7.64 (d, J = 8.8 Hz, 2H); ^{13}C NMR (126 MHz, $CDCl_3$): δ 147.2, 142.1, 132.2, 126.6, 125.2, 122.7.

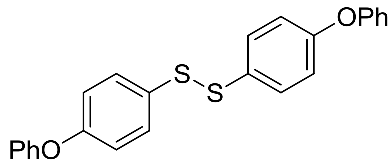

1,2-Bis(4-phenoxyphenyl)disulfane (**3k**) [11]

70%; yellow liquid. ^{1}H NMR (500 MHz, $CDCl_3$): δ 7.47 (d, J = 8.8 Hz, 4H), 7.36 (t, J = 8.3 Hz , 4H), 7.15 (t, J = 7.9 Hz, 2H), 7.03 (d, J = 8.7 Hz, 4H), 6.95 (d, J = 8.8 Hz, 4H); ^{13}C NMR (126 MHz, $CDCl_3$): δ 157.7, 156.6, 133.3, 131.4, 129.9, 123.9, 119.4, 119.1.

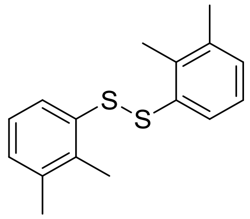

1,2-Bis(2,3-dimethylphenyl)disulfane (**3l**) [12]

76%; white solid. ^{1}H NMR (500 MHz, $CDCl_3$): δ 7.41 (d, J = 7.0 Hz, 2H), 7.03 (m, 4H), 2.37 (s, 6H), 2.30 (s, 6H); ^{13}C NMR (126 MHz, $CDCl_3$): δ 137.5, 135.9, 135.8, 129.0, 126.8, 126.1, 20.4, 16.3.

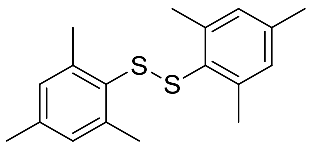

1,2-Dimesityldisulfane (**3m**) [13]

56%; white solid. ^{1}H NMR (500 MHz, $CDCl_3$): δ 6.84 (s, 4H), 2.26 (s, 6H), 2.21 (s, 12H); ^{13}C NMR (126 MHz, $CDCl_3$): δ 143.4, 139.4, 131.7, 129.0, 21.5, 21.2.

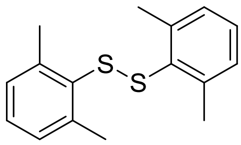

1,2-Bis(2,6-dimethylphenyl)disulfane (**3n**) [14]

42%; white solid. ^{1}H NMR (500 MHz, $CDCl_3$): δ 7.11 (t, J = 7.5 Hz, 2H), 7.02 (d, J = 7.5 Hz, 4H), 2.25 (s, 12H); ^{13}C NMR (126 MHz, $CDCl_3$): δ 143.6, 134.9, 129.4, 128.2, 21.6.

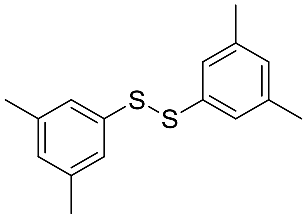

1,2-Bis(3,5-dimethylphenyl)disulfane (**3o**) [15]

56%; white solid. ^{1}H NMR (500 MHz, $CDCl_3$): δ 7.13(s, 4H), 6.86 (s, 2H), 2.29 (s, 12H); ^{13}C NMR (126 MHz, $CDCl_3$): δ 138.9, 137.0, 129.2, 125.3, 21.4.

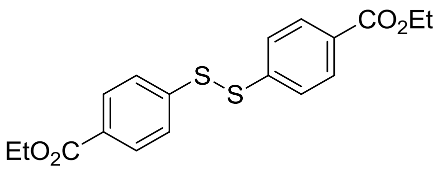

Diethyl 4,4'-disulfanediyldibenzoate (**3p**) [16]

92%; white solid. ^{1}H NMR (500 MHz, $CDCl_3$): δ 7.97 (d, J = 8.7 Hz, 2H), 7.52 (d, J = 8.6 Hz, 2H), 4.35 (q, J = 7.1 Hz, 2H), 1.37 (t, J = 7.1 Hz, 3H); ^{13}C NMR (126 MHz, $CDCl_3$): δ 166.0, 142.1, 130.3, 129.3, 126.1, 61.2, 14.4.

References

(1) Qin, H.-L.; Zheng, Q.; Bare, G. A. L.; Wu, P.; Sharpless, K. B. *Angew. Chem. Int. Ed.* **2016**, *55*, 14155-14158.

(2) Banfield, S. C.; Omori, A. T.; Leisch, H.; Hudlicky, T.; *J. Org. Chem.* **2007**, *72*, 4989-4992.

(3) Vandavasi, J. K.; Hu, W.-P.; Chen, C.-Y.; Wang, J.-J.; *Tetrahedron,* **2011**, *67*, 8895-8901.

(4) Zhao, J.-W.; Song, T.; Jiang, H.-L.; Xu, L.-Z.; Zhu, S.-Z.; *Chin. J. Chem.* **2010**, *28*, 1623-1629.

(5) Khan, K. M.; Taha, M.; Rahim, F.; Ali, M.; Jamil, W.; Perveen, S.; Choudhary, M. I.; *Lett. Org. Chem.* **2010**, *7,* 415-419.

(6) Hunter. W. H.; Kohlhase, H.; *J. Am. Chem. Soc.* **1932**, *54*, 2425-2432.

(7) Srisook, E.; Chi, D. Y.; *Bull. Korean Chem. Soc.* **2004**, *25*, 895-899.

(8) Soleiman-Beigi, M.; Arzehgar, Z.; *Monatsh Chem*. **2016**, doi: 10.1007/s00706-016-1673-2

(9) Misra, A. K.; Agnihotri, G.; *Synthetic Commun.* **2004**, *34*, 1079-1085.

(10) Riegel, B.; Lappin, G. R.; Adelson, B. H.; Jackson, R. I.; Albisetti, C. J.; Dodson, J. R. M.; Baker, R. H.; *J. Am. Chem. Soc.* **1946**, *68*, 1264-1266.

(11) Suter, C. M.; *J. Am. Chem. Soc.* **1931**, *53*, 1112-1116.

(12) Bhar, D.; Chandrasekaran, S.; *Synthesis*, **1994**, *8*, 785-786.

(13) Suziki, H.; Shinoda, M.; *B. Chem. Soc. Jpn.* **1977**, *50*, 321-322.

(14) Ruano, J. L. G.; Parra, A.; Aleman, J. *Green Chem*. **2008**, *10*, 706–711.

(15) Spurg, A.; Schnakenburg, G.; Waldvogel, S. R.; *Chem. Eur. J.* **2009**, *15*, 13313-13317.

(16) Meier, H.; Konnerth, U.; Graw, S.; Echter, T.; *Chem. Ber.* **1984**, *117*, 107-126.

4. NMR spectra

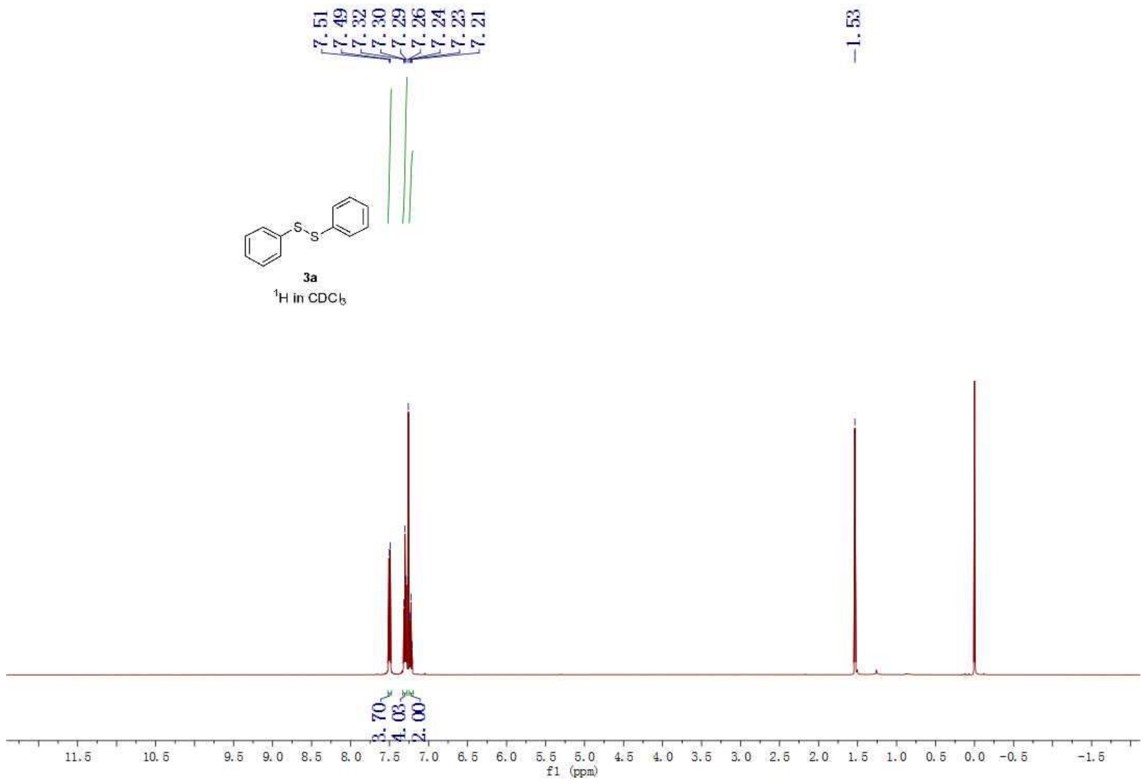

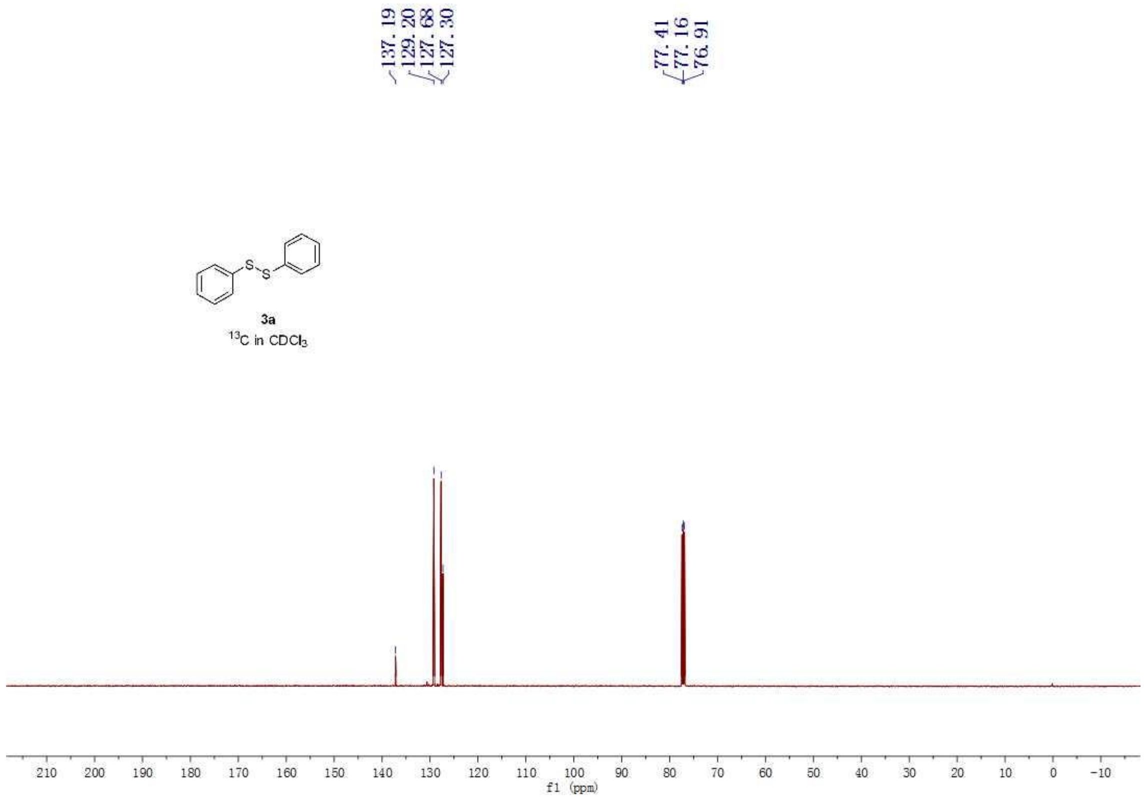

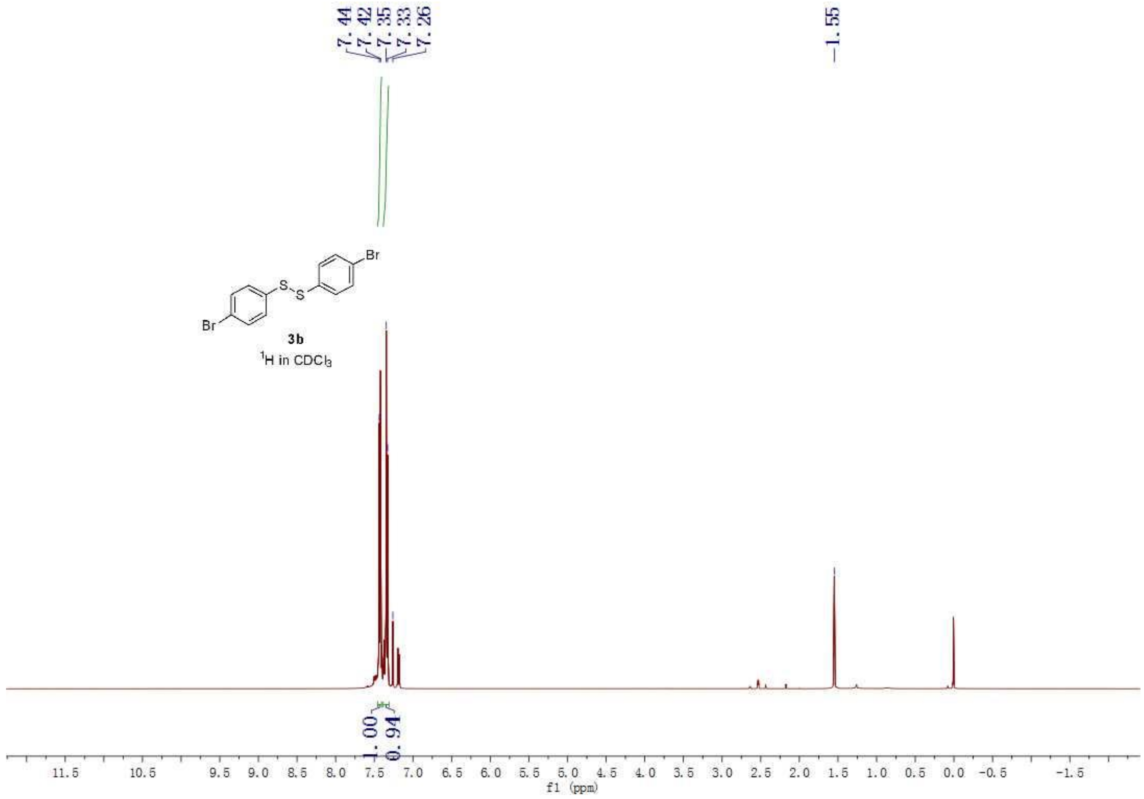
7.44
7.42
7.35
7.33
7.26
1.55
Br
S
S
Br
3b
1H in CDCl3
1.00
0.94
11.5 10.5 9.5 9.0 8.5 8.0 7.5 7.0 6.5 6.0 5.5 5.0 4.5 4.0 3.5 3.0 2.5 2.0 1.5 1.0 0.5 0.0 -0.5 -1.5
f1 (ppm)

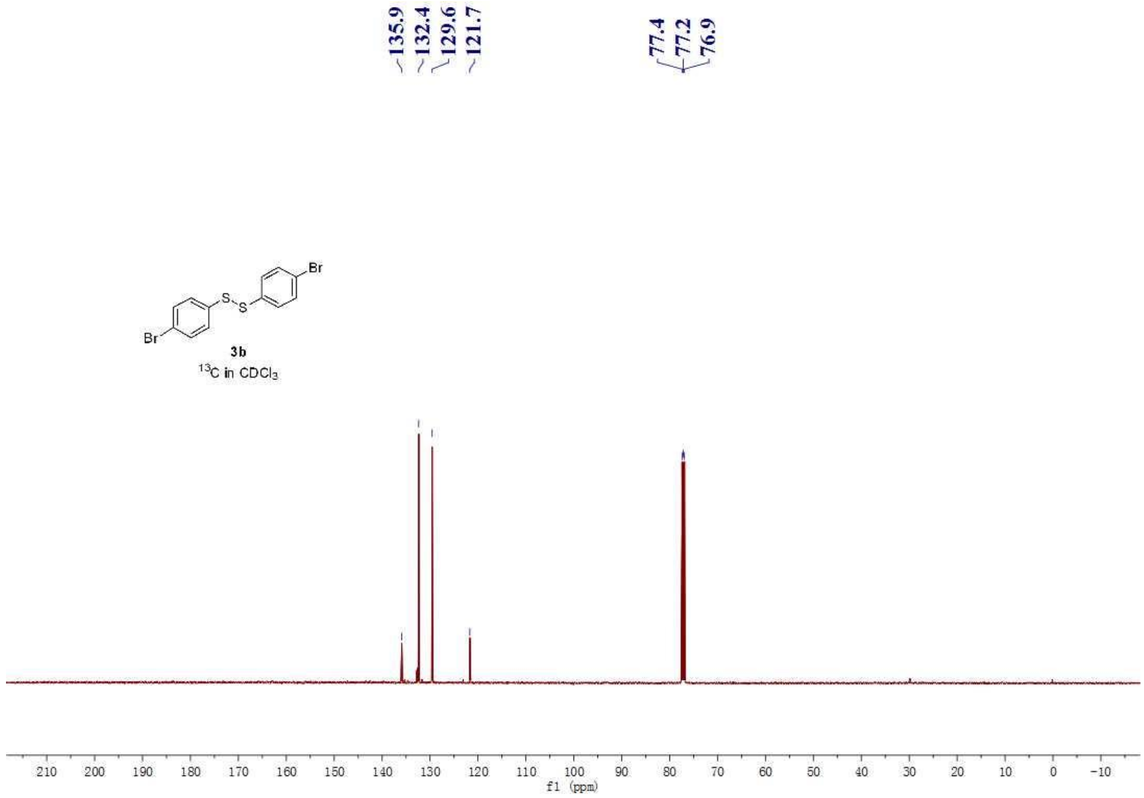
135.9
132.4
129.6
121.7
77.4
77.2
76.9
Br
S
S
Br
3b
13C in CDCl3
210 200 190 180 170 160 150 140 130 120 110 100 90 80 70 60 50 40 30 20 10 0 -10
f1 (ppm)

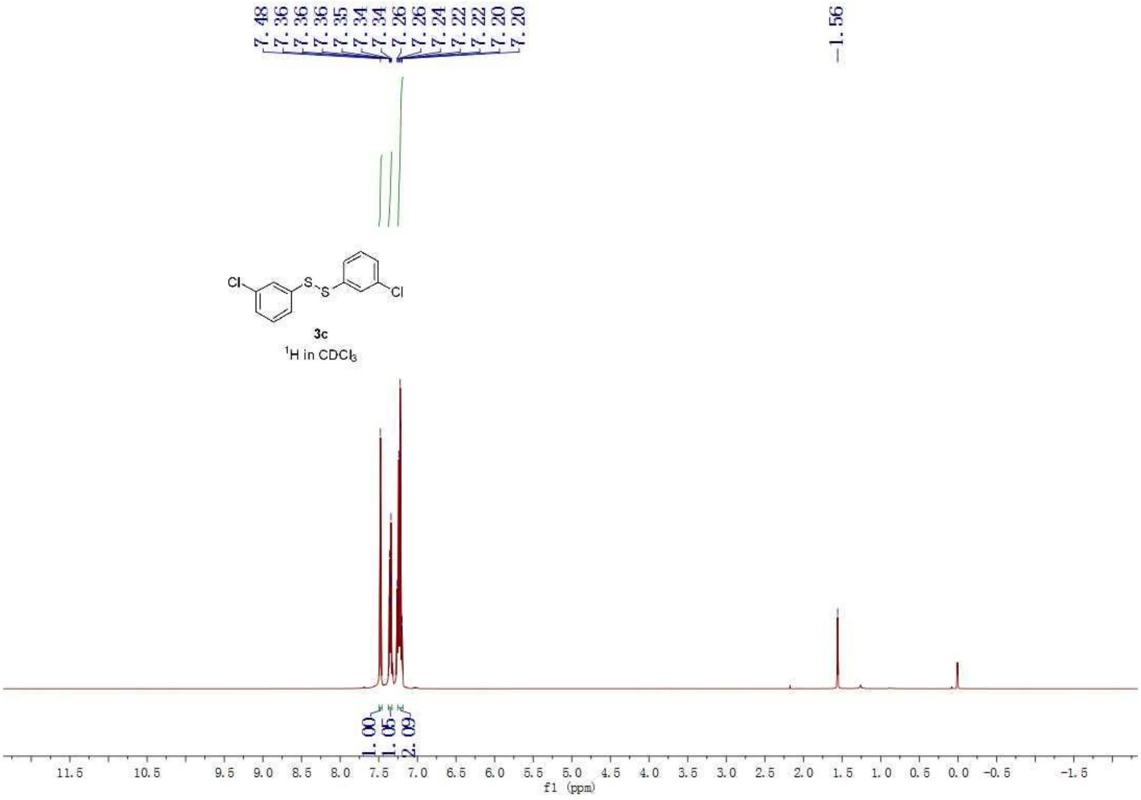
7.48
7.36
7.36
7.36
7.35
7.34
7.34
7.26
7.26
7.24
7.22
7.22
7.20
7.20
1.56
Cl
S
S
Cl
3c
¹H in CDCl₃
1.00
1.05
2.09
f1 (ppm)

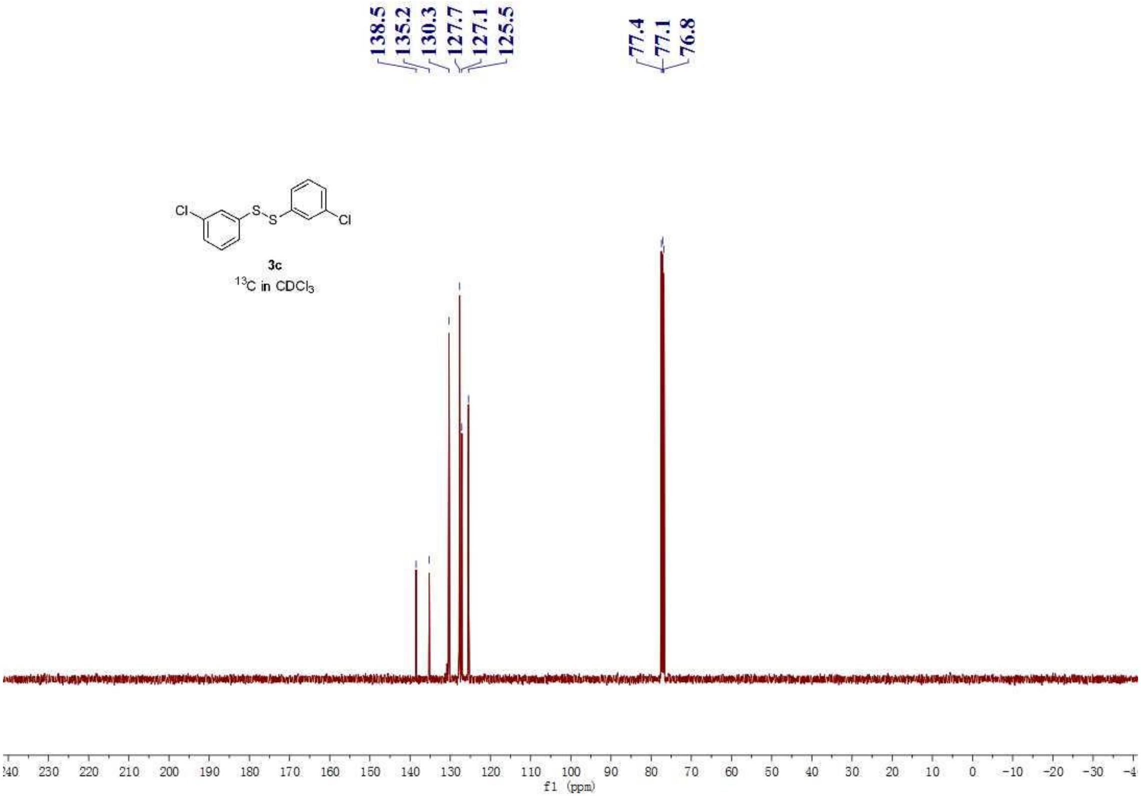
138.5
135.2
130.3
127.7
127.1
125.5
77.4
77.1
76.8
Cl
S
S
Cl
3c
¹³C in CDCl₃
f1 (ppm)

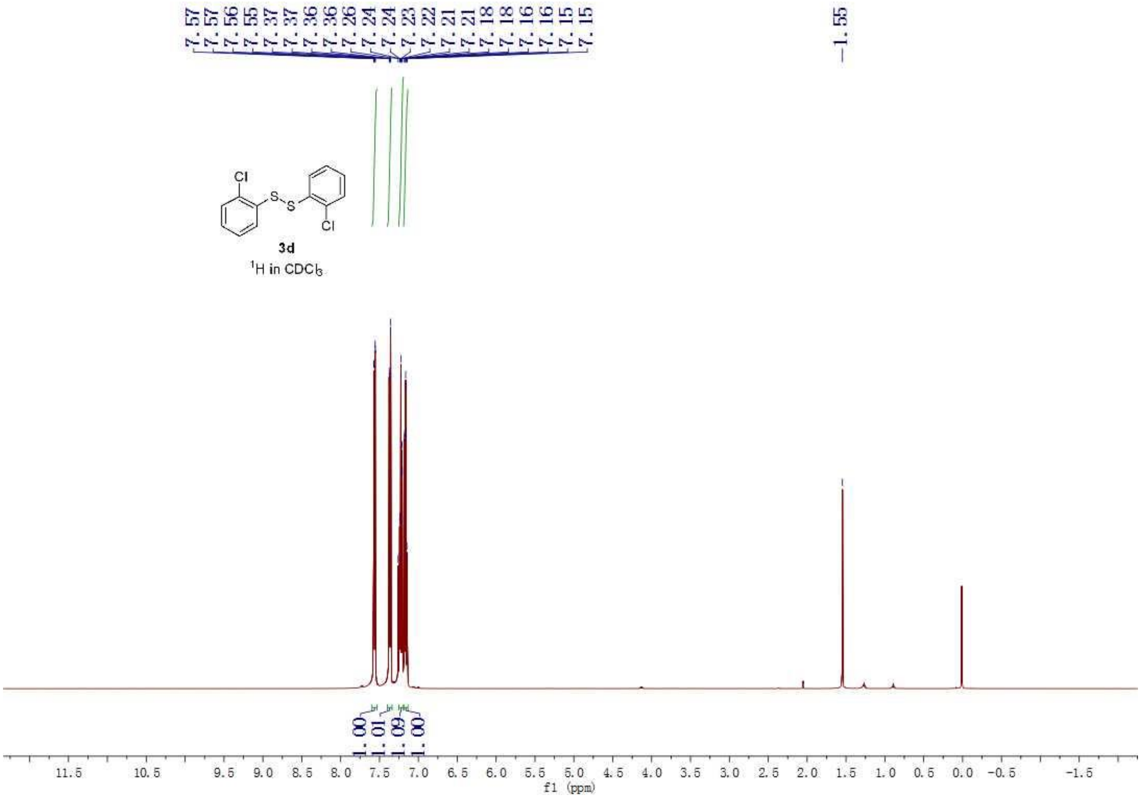
7.57
7.57
7.56
7.55
7.37
7.37
7.36
7.36
7.26
7.24
7.24
7.23
7.22
7.21
7.21
7.18
7.18
7.16
7.15
7.15
1.55
3d
1H in CDCl3
1.00
1.01
1.09
1.00
11.5 10.5 9.5 9.0 8.5 8.0 7.5 7.0 6.5 6.0 5.5 5.0 4.5 4.0 3.5 3.0 2.5 2.0 1.5 1.0 0.5 0.0 -0.5 -1.5
f1 (ppm)

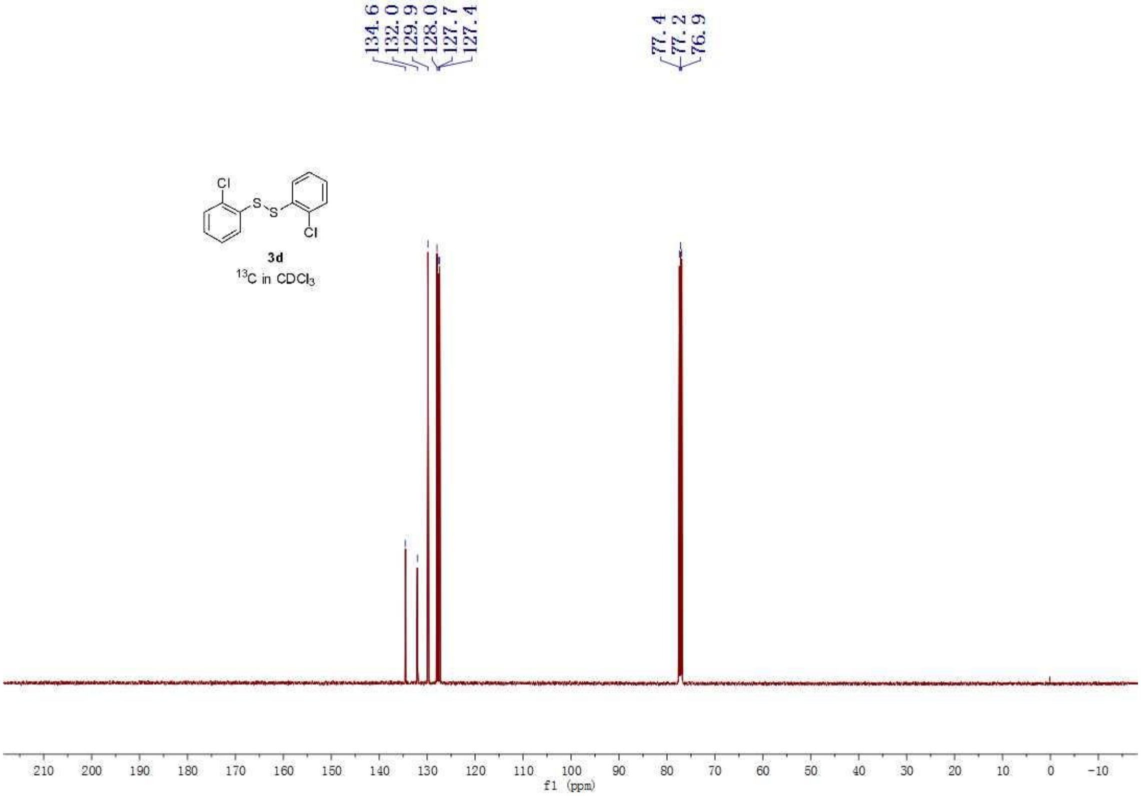
134.6
132.0
129.9
128.0
127.7
127.4
77.4
77.2
76.9
3d
13C in CDCl3
210 200 190 180 170 160 150 140 130 120 110 100 90 80 70 60 50 40 30 20 10 0 -10
f1 (ppm)

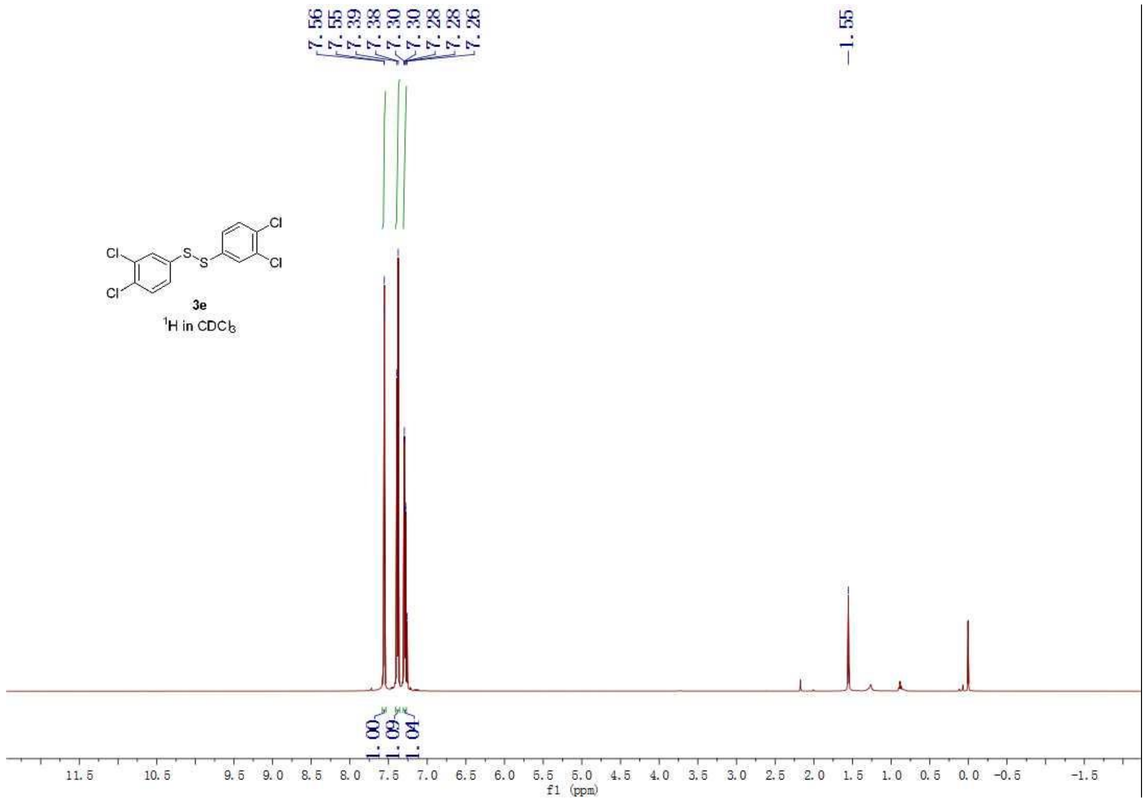
7.56
7.55
7.39
7.38
7.30
7.30
7.28
7.28
7.26
1.55
Cl
S
S
Cl
Cl
Cl
3e
1H in CDCl3
1.00
1.09
1.04
11.5 10.5 9.5 9.0 8.5 8.0 7.5 7.0 6.5 6.0 5.5 5.0 4.5 4.0 3.5 3.0 2.5 2.0 1.5 1.0 0.5 0.0 -0.5 -1.5
f1 (ppm)

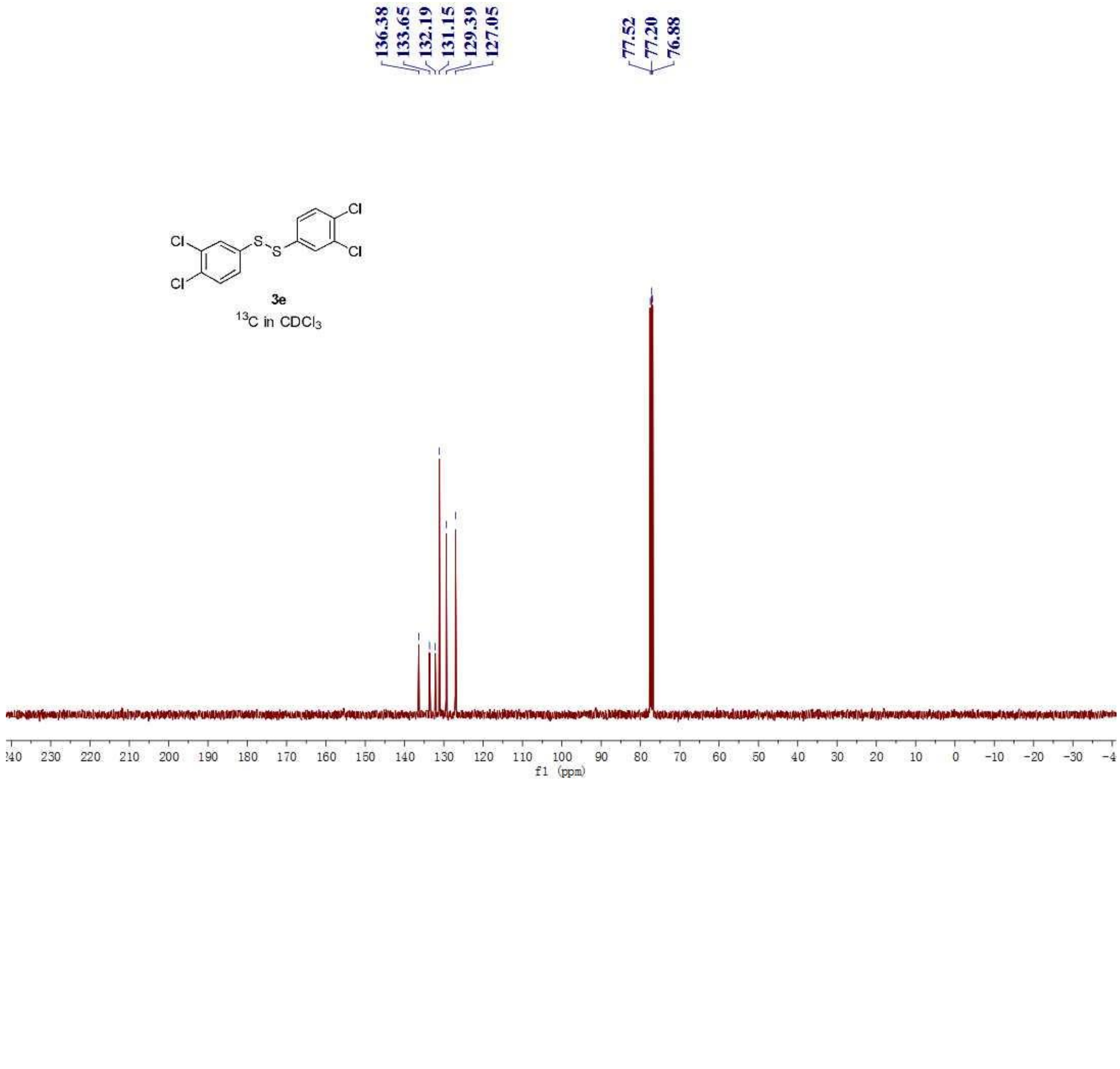
136.38
133.65
132.19
131.15
129.39
127.05
77.52
77.20
76.88
Cl
S
S
Cl
Cl
Cl
3e
13C in CDCl3
240 230 220 210 200 190 180 170 160 150 140 130 120 110 100 90 80 70 60 50 40 30 20 10 0 -10 -20 -30 -4
f1 (ppm)

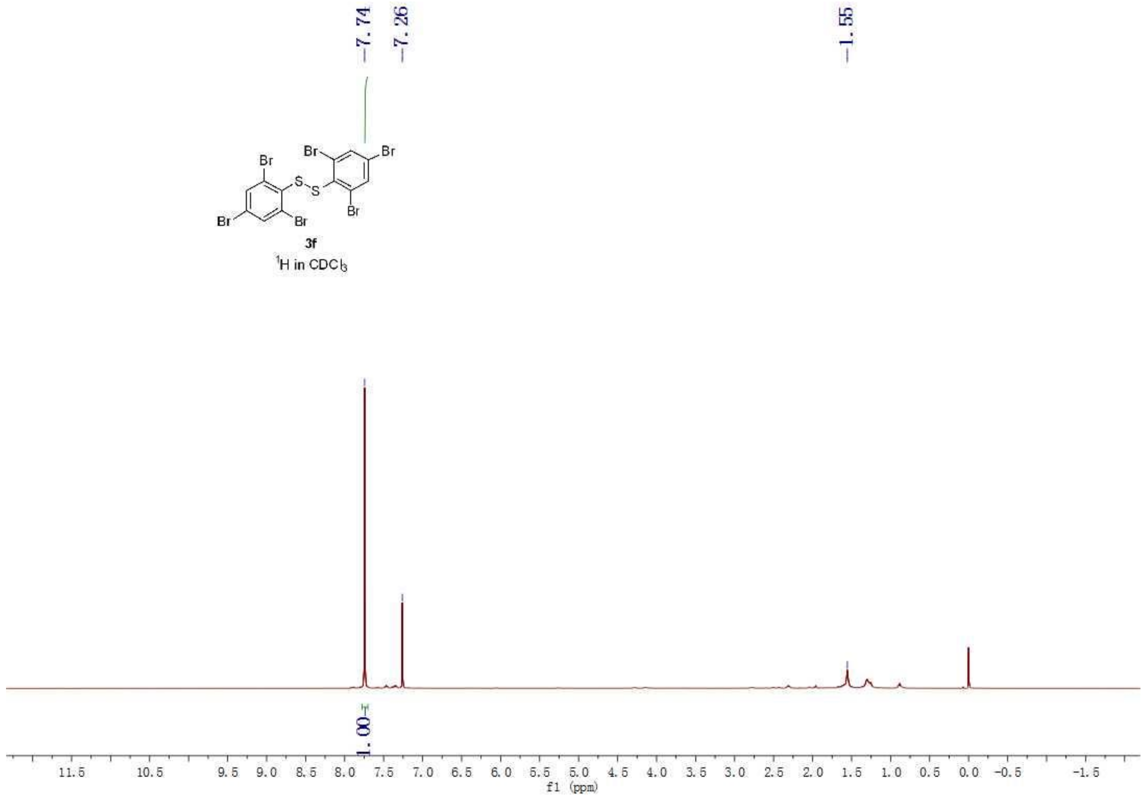

7.74
7.26
1.55
Br
S
S
3f
1H in CDCl3
1.00
11.5
10.5
9.5
9.0
8.5
8.0
7.5
7.0
6.5
6.0
5.5
5.0
4.5
4.0
3.5
3.0
2.5
2.0
1.5
1.0
0.5
0.0
-0.5
-1.5
f1 (ppm)

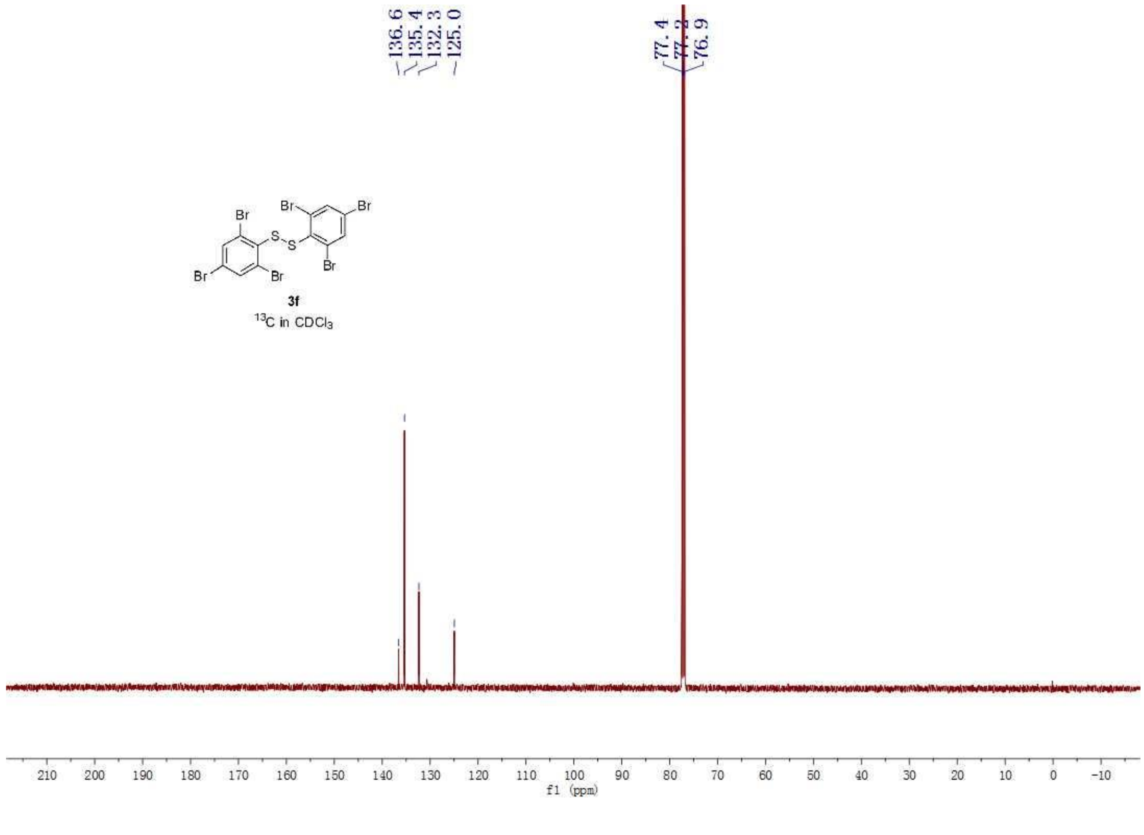

136.6
135.4
132.3
125.0
77.4
77.2
76.9
Br
S
S
3f
13C in CDCl3
210
200
190
180
170
160
150
140
130
120
110
100
90
80
70
60
50
40
30
20
10
0
-10
f1 (ppm)

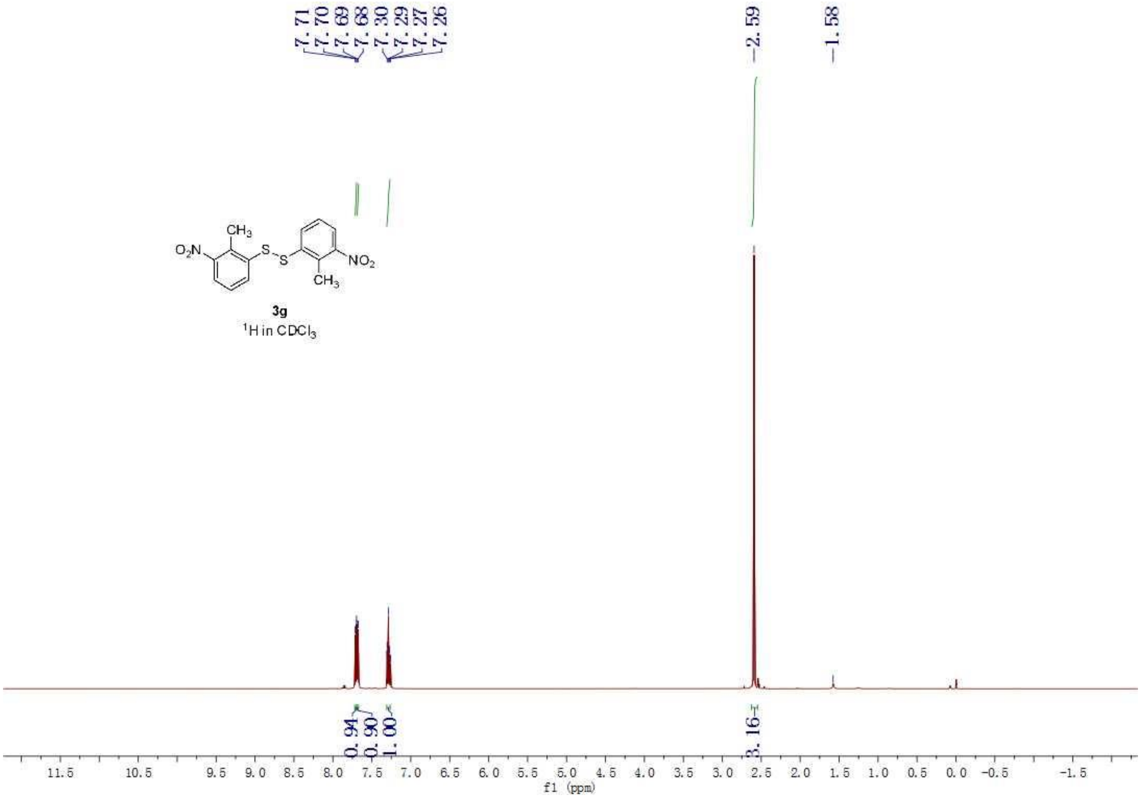

7.71
7.70
7.69
7.68
7.30
7.29
7.27
7.26
2.59
1.58
3g
^{1}H in CDCl$_3$
0.94
0.90
1.00
3.16
11.5 10.5 9.5 9.0 8.5 8.0 7.5 7.0 6.5 6.0 5.5 5.0 4.5 4.0 3.5 3.0 2.5 2.0 1.5 1.0 0.5 0.0 -0.5 -1.5
f1 (ppm)

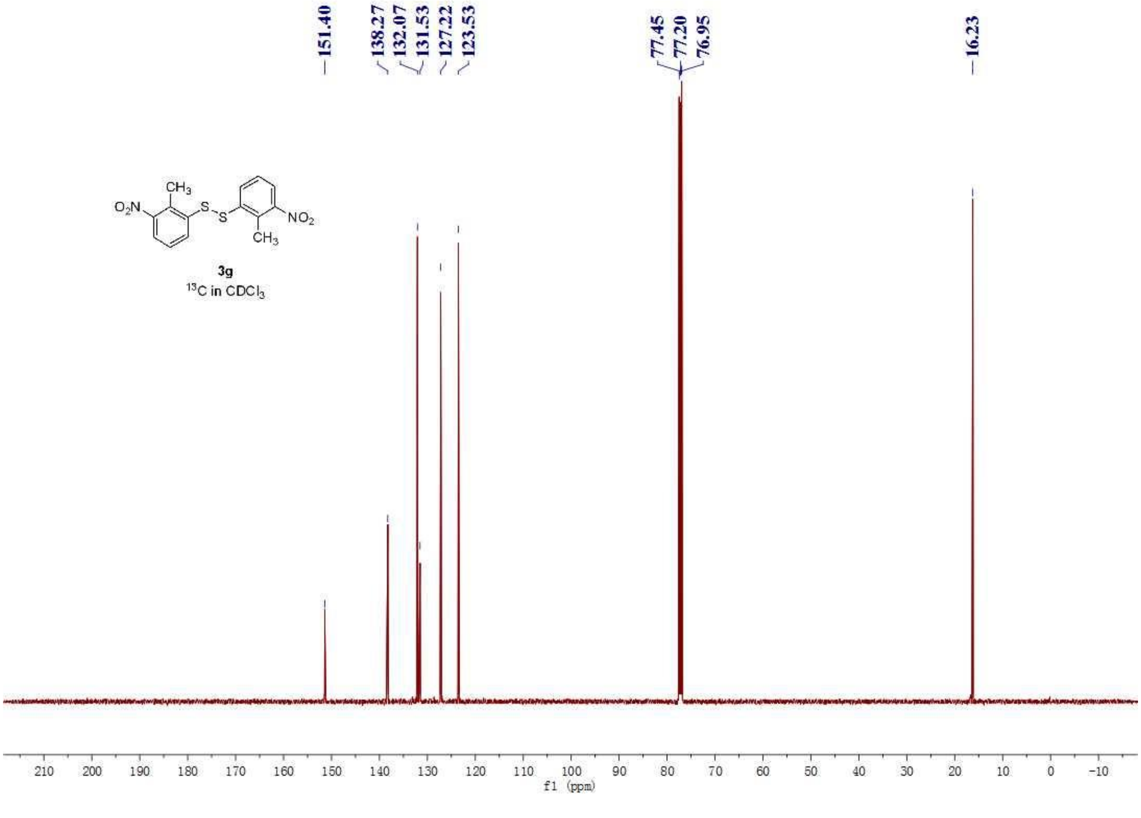

151.40
138.27
132.07
131.53
127.22
123.53
77.45
77.20
76.95
16.23
3g
^{13}C in CDCl$_3$
210 200 190 180 170 160 150 140 130 120 110 100 90 80 70 60 50 40 30 20 10 0 -10
f1 (ppm)

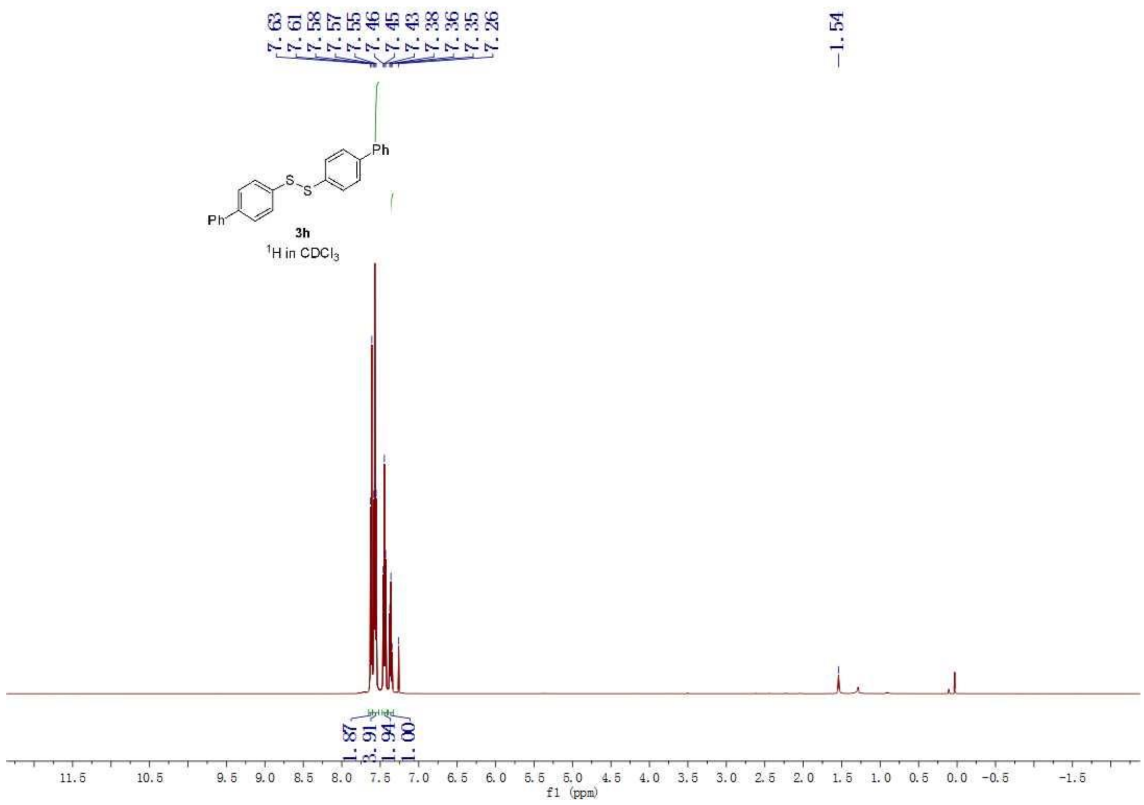
7.63
7.61
7.58
7.57
7.55
7.46
7.45
7.43
7.38
7.36
7.35
7.26
1.54
Ph
S
S
Ph
3h
^{1}H in $CDCl_3$
1.87
3.91
1.94
1.00
11.5
10.5
9.5
9.0
8.5
8.0
7.5
7.0
6.5
6.0
5.5
5.0
4.5
4.0
3.5
3.0
2.5
2.0
1.5
1.0
0.5
0.0
-0.5
-1.5
f1 (ppm)

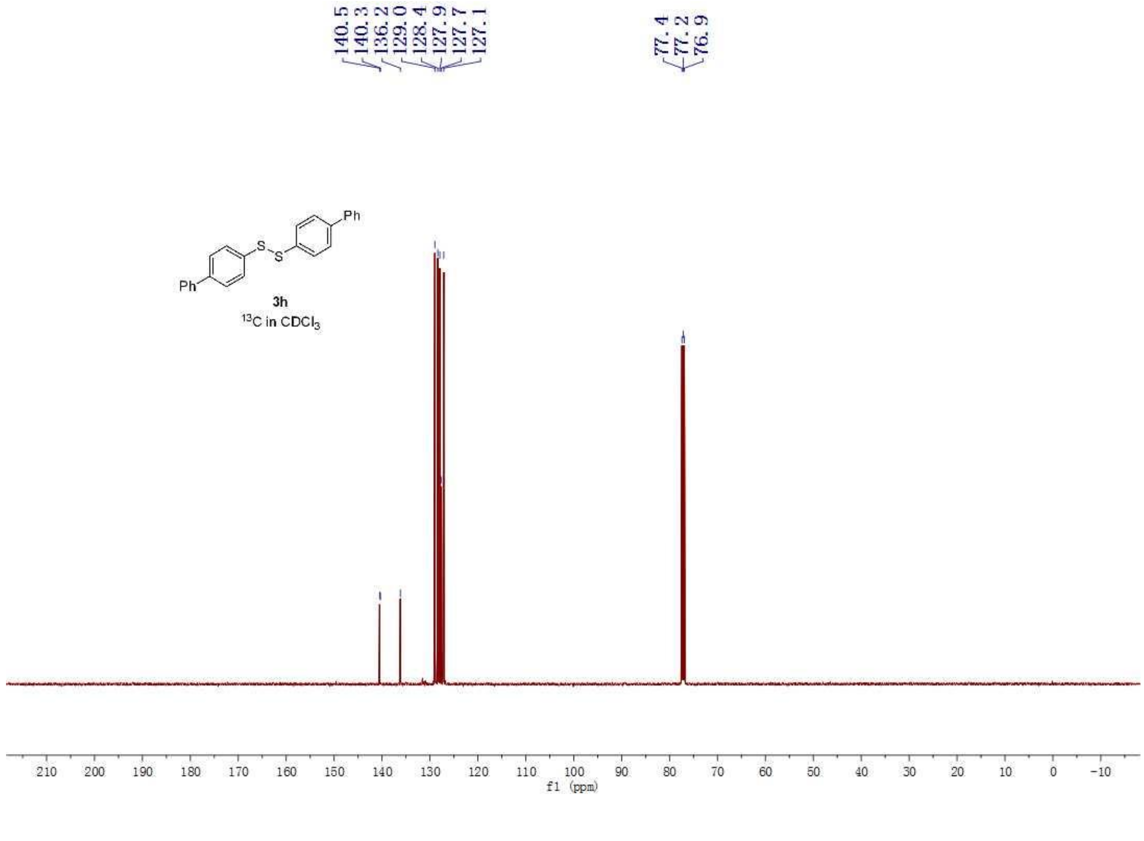
140.5
140.3
136.2
129.0
128.4
127.9
127.7
127.1
77.4
77.2
76.9
Ph
S
S
Ph
3h
^{13}C in $CDCl_3$
210
200
190
180
170
160
150
140
130
120
110
100
90
80
70
60
50
40
30
20
10
0
-10
f1 (ppm)

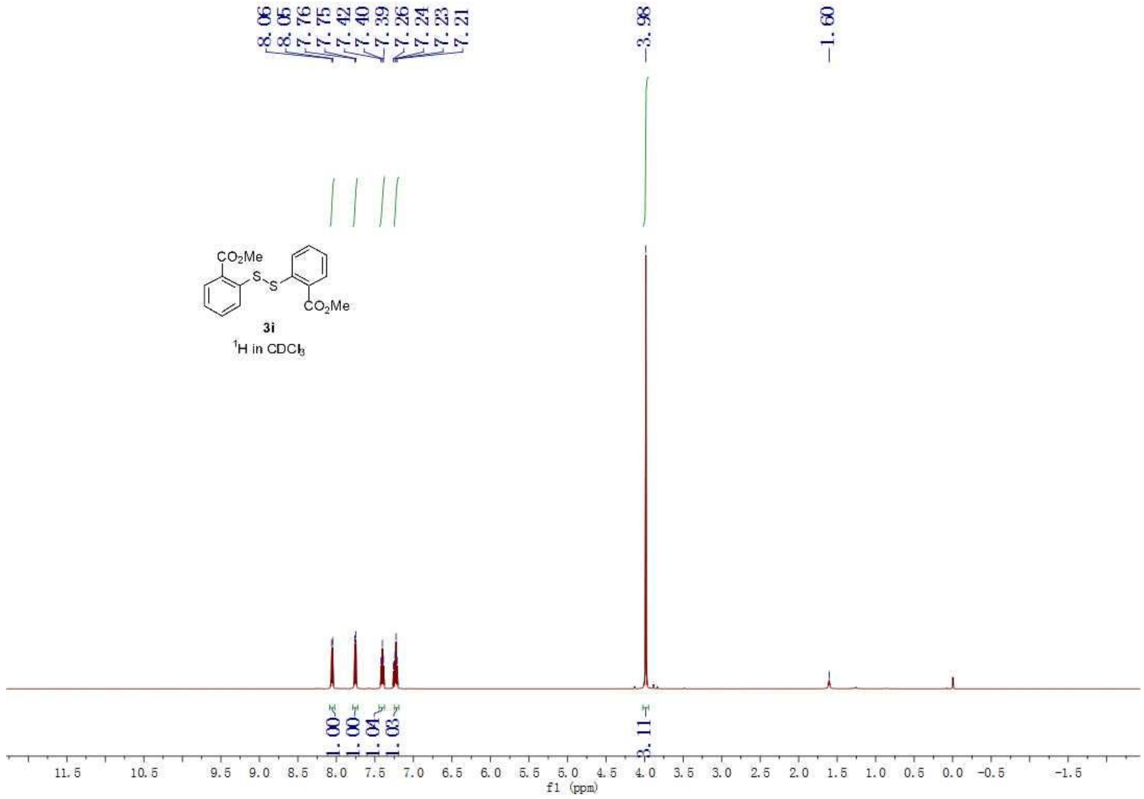
8.06
8.05
7.76
7.75
7.42
7.40
7.39
7.26
7.24
7.23
7.21
3.98
1.60
CO2Me
S
S
CO2Me
3i
1H in CDCl3
1.00
1.00
1.04
1.03
3.11
f1 (ppm)

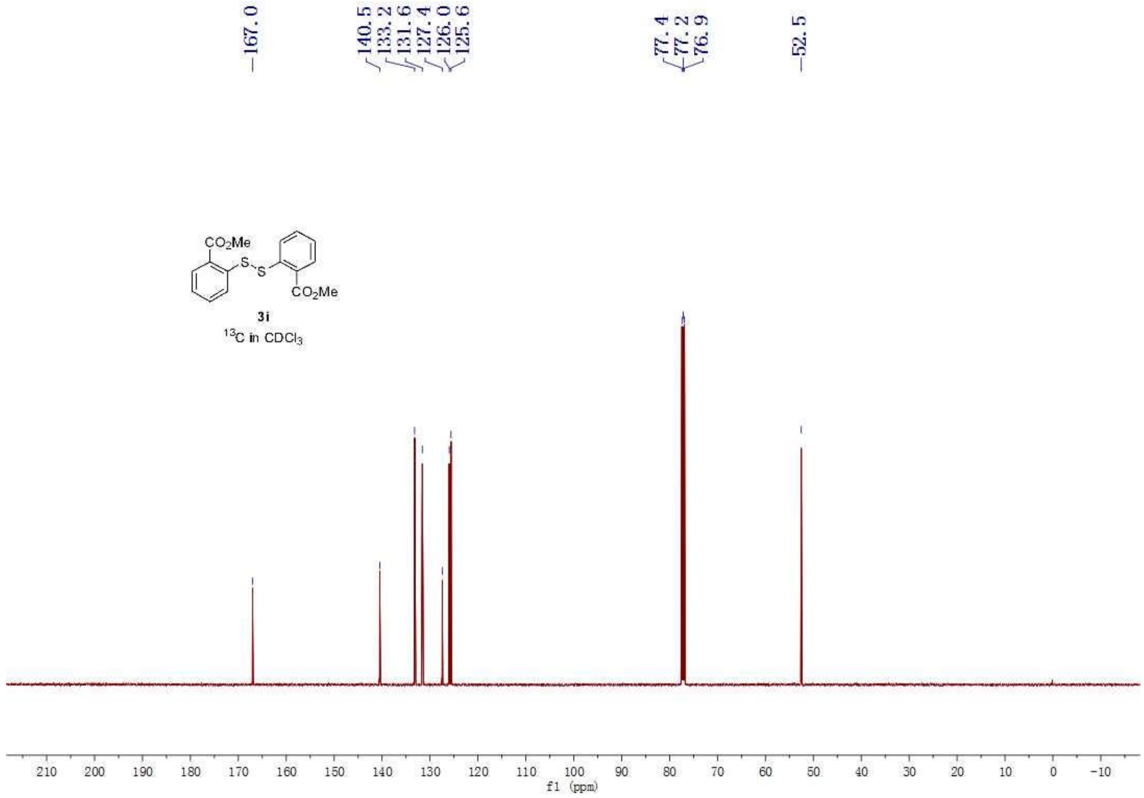
167.0
140.5
133.2
131.6
127.4
126.0
125.6
77.4
77.2
76.9
52.5
CO2Me
S
S
CO2Me
3i
13C in CDCl3
f1 (ppm)

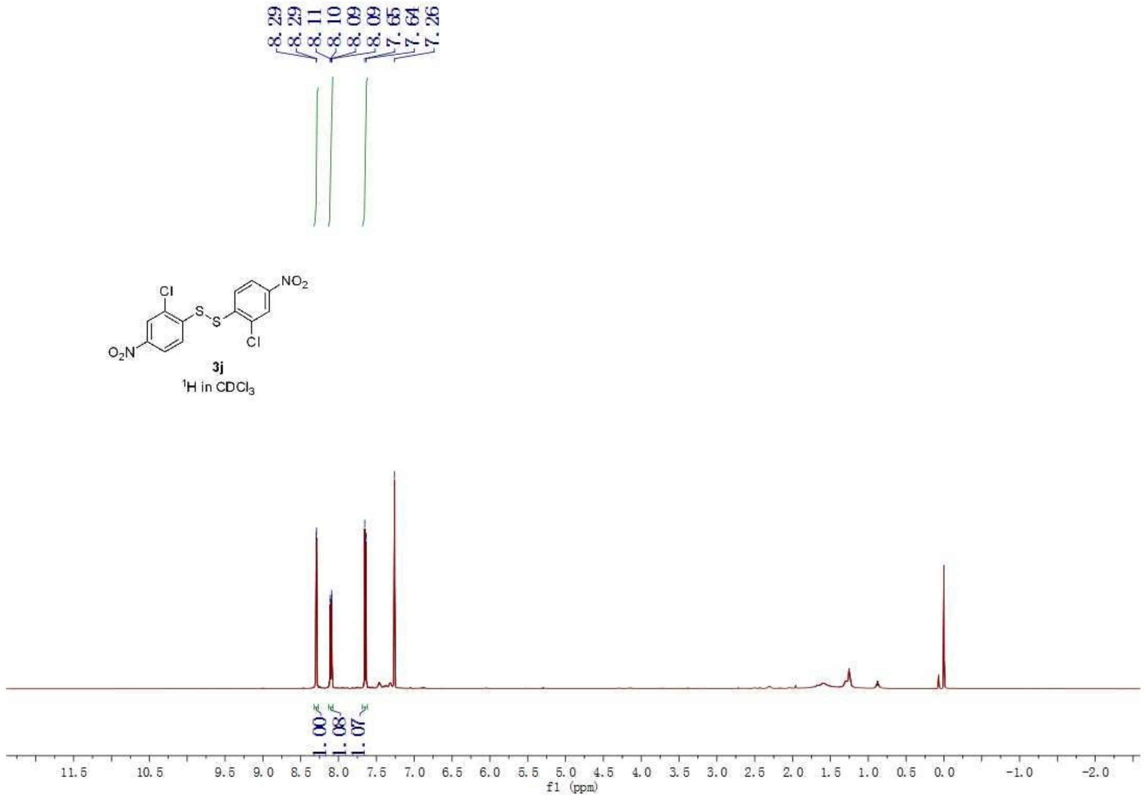
8.29
8.29
8.11
8.10
8.09
8.09
7.65
7.64
7.26
3j
1H in CDCl3
1.00
1.08
1.07
f1 (ppm)

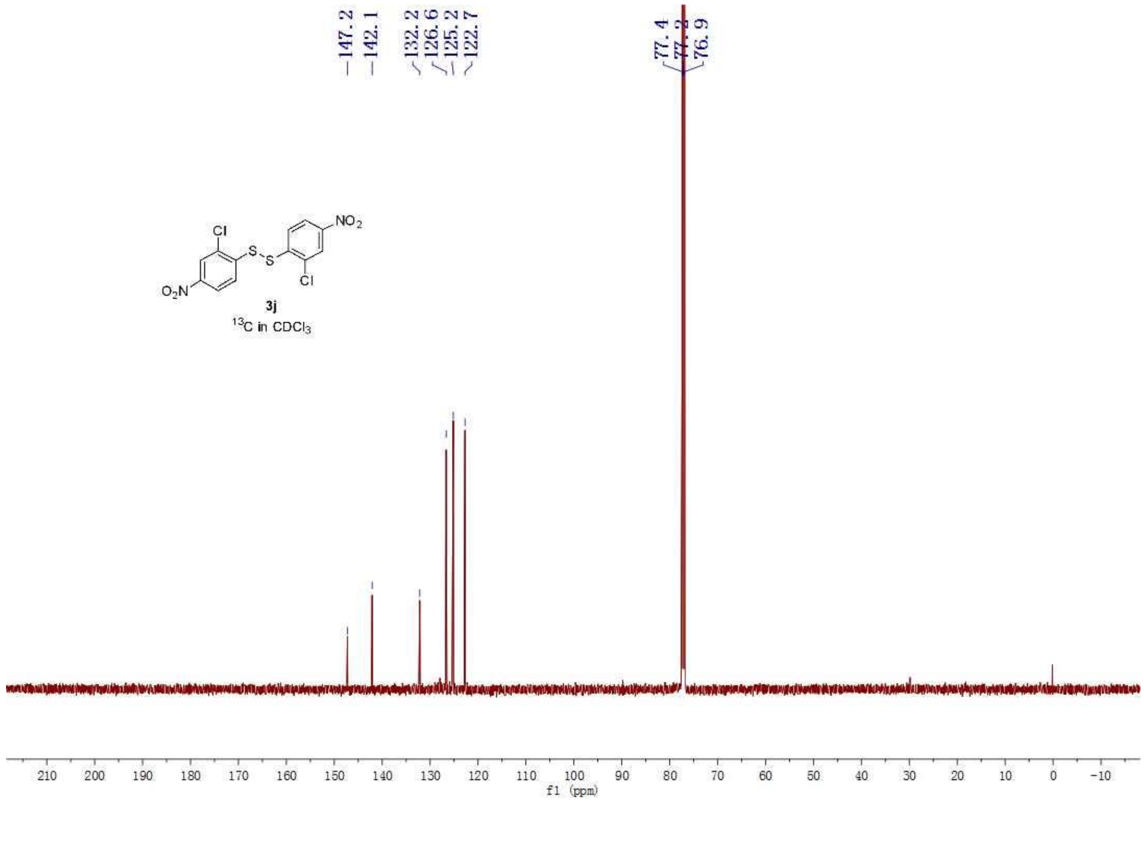
147.2
142.1
132.2
126.6
125.2
122.7
77.4
77.2
76.9
3j
13C in CDCl3
f1 (ppm)

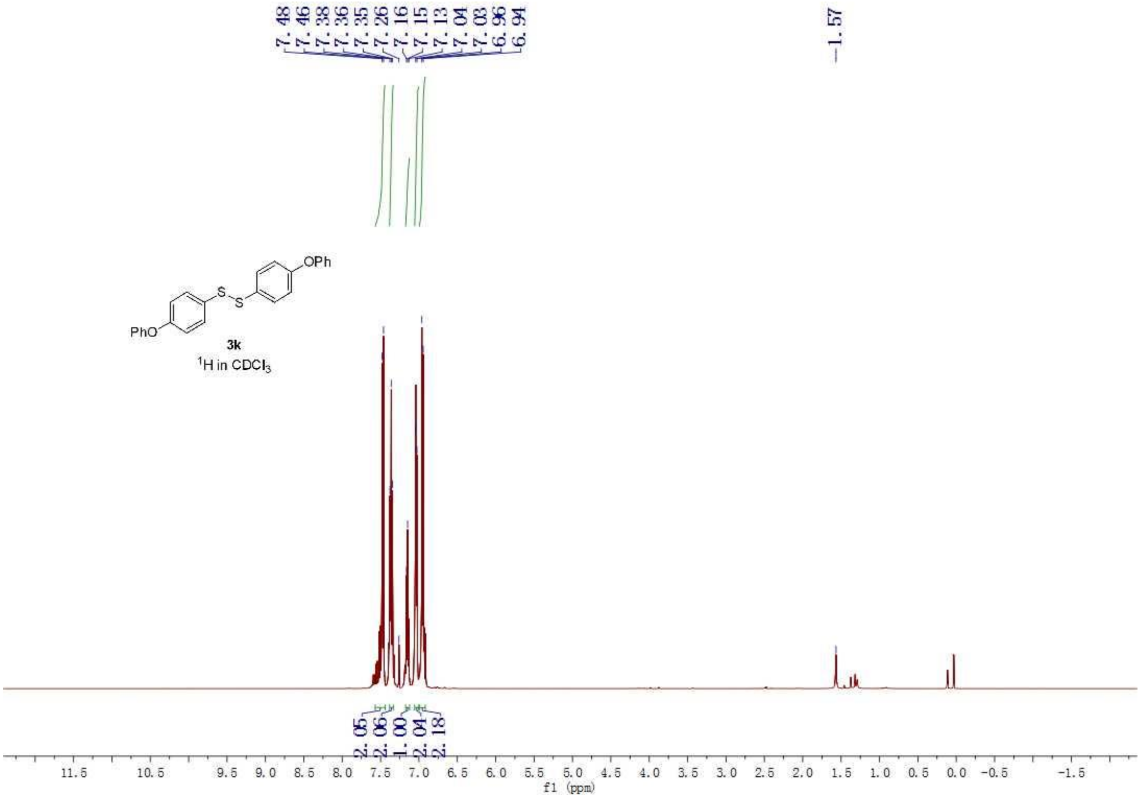
7.48
7.46
7.38
7.36
7.35
7.26
7.16
7.15
7.13
7.04
7.03
6.96
6.94
1.57
OPh
PhO
S
S
3k
1H in CDCl3
2.05
2.06
1.00
2.04
2.18
f1 (ppm)

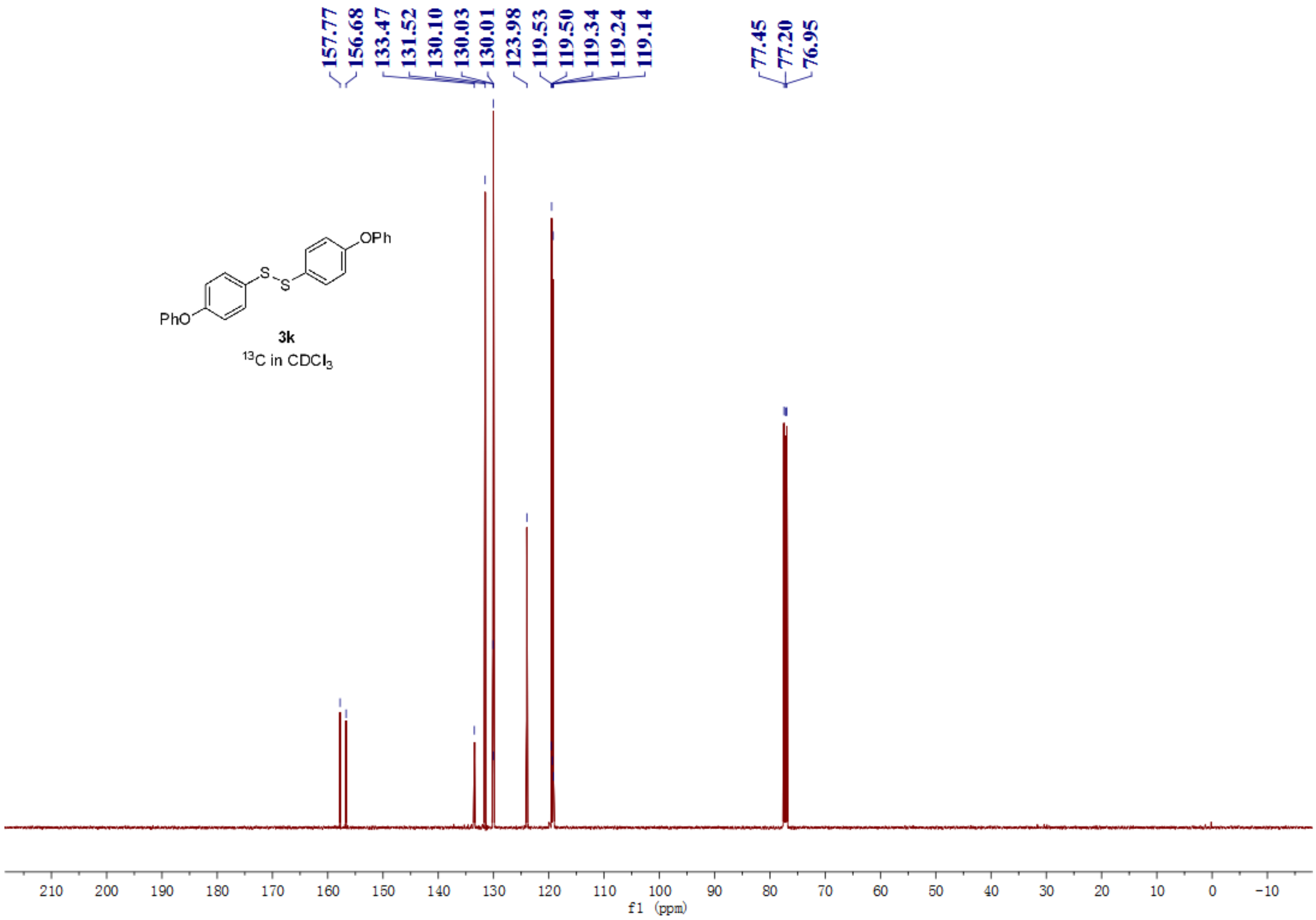
157.77
156.68
133.47
131.52
130.10
130.03
130.01
123.98
119.53
119.50
119.34
119.24
119.14
77.45
77.20
76.95
OPh
PhO
S
S
3k
13C in CDCl3
f1 (ppm)

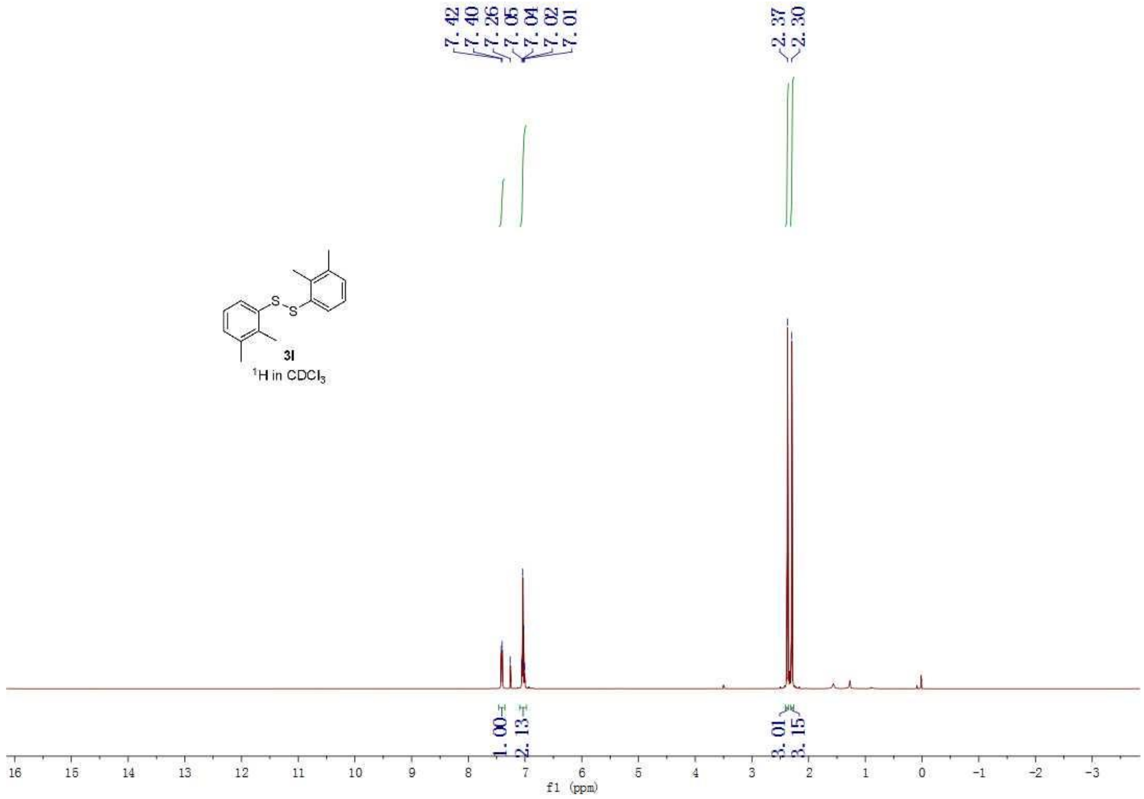
7.42
7.40
7.26
7.05
7.04
7.02
7.01
2.37
2.30
3l
^{1}H in $CDCl_3$
1.00
2.13
3.01
3.15
16 15 14 13 12 11 10 9 8 7 6 5 4 3 2 1 0 -1 -2 -3
f1 (ppm)

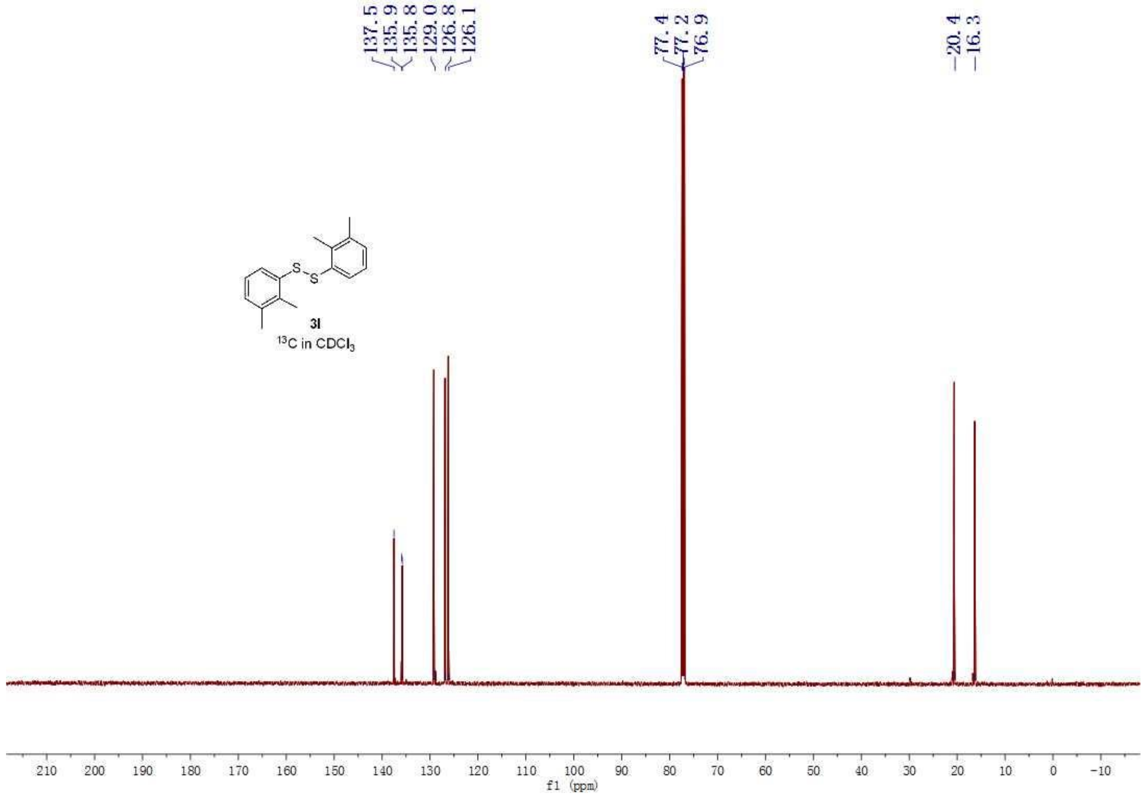
137.5
135.9
135.8
129.0
126.8
126.1
77.4
77.2
76.9
20.4
16.3
3l
^{13}C in $CDCl_3$
210 200 190 180 170 160 150 140 130 120 110 100 90 80 70 60 50 40 30 20 10 0 -10
f1 (ppm)

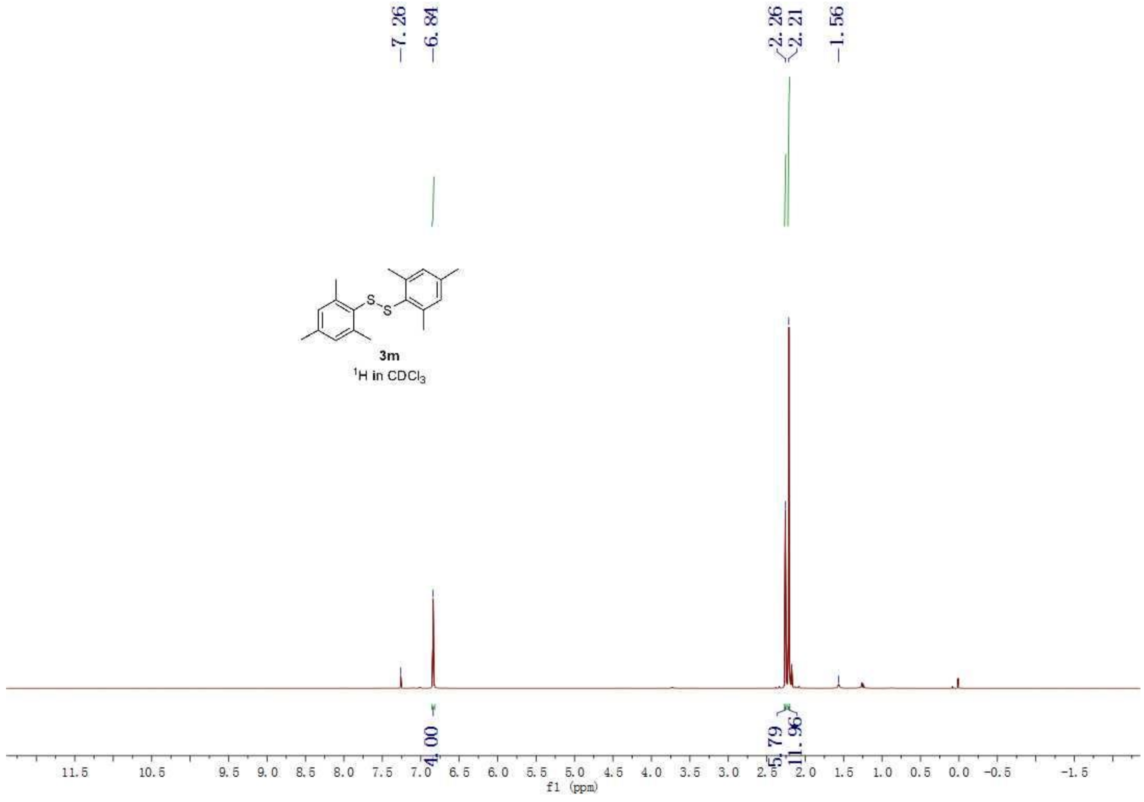

7.26
6.84
2.26
2.21
1.56
3m
^{1}H in $CDCl_3$
4.00
5.79
11.96
11.5
10.5
9.5 9.0 8.5 8.0 7.5 7.0 6.5 6.0 5.5 5.0 4.5 4.0 3.5 3.0 2.5 2.0 1.5 1.0 0.5 0.0 -0.5
-1.5
f1 (ppm)

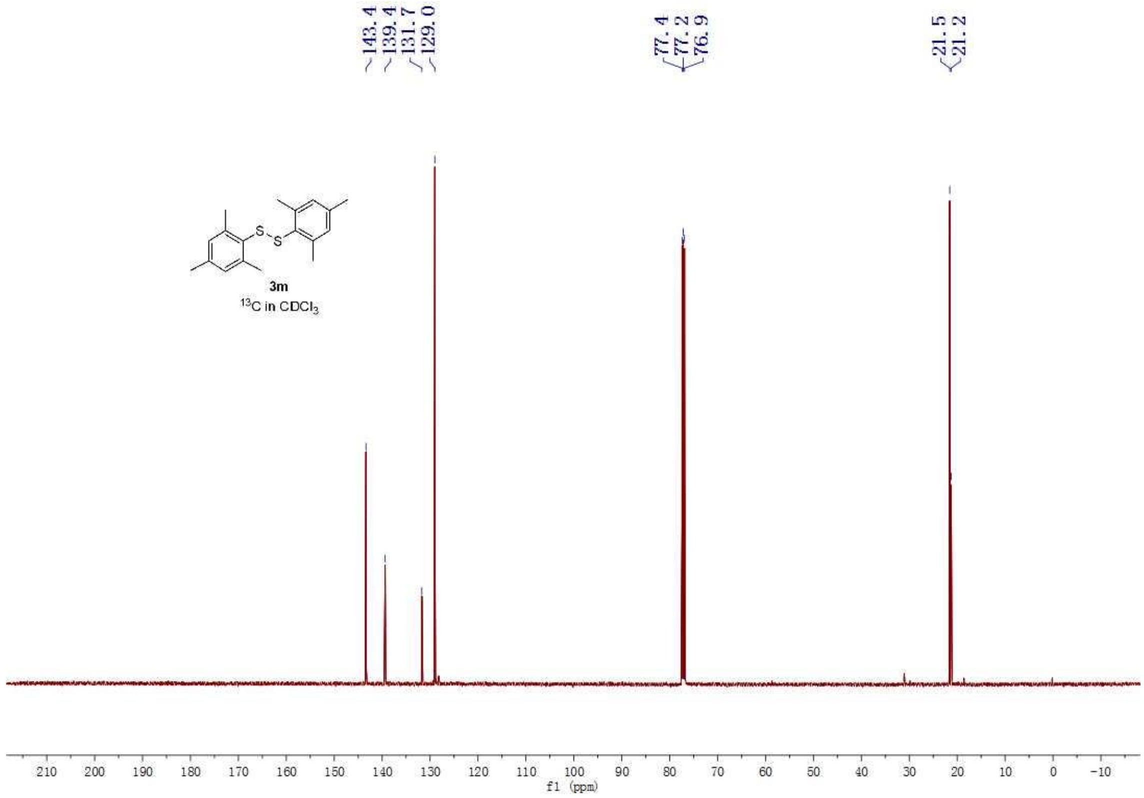

143.4
139.4
131.7
129.0
77.4
77.2
76.9
21.5
21.2
3m
^{13}C in $CDCl_3$
210 200 190 180 170 160 150 140 130 120 110 100 90 80 70 60 50 40 30 20 10 0 -10
f1 (ppm)

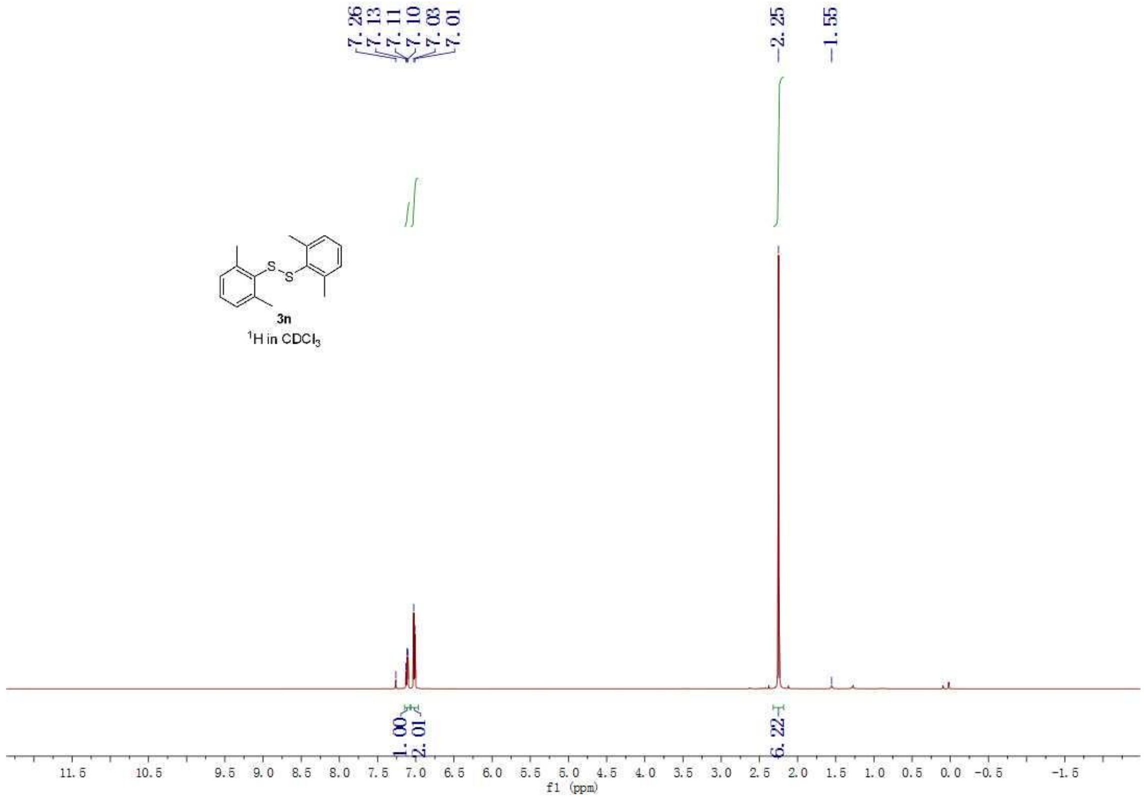
7.26
7.13
7.11
7.10
7.03
7.01
2.25
1.55
3n
1H in CDCl3
1.00
2.01
6.22
11.5 10.5 9.5 9.0 8.5 8.0 7.5 7.0 6.5 6.0 5.5 5.0 4.5 4.0 3.5 3.0 2.5 2.0 1.5 1.0 0.5 0.0 -0.5 -1.5
f1 (ppm)

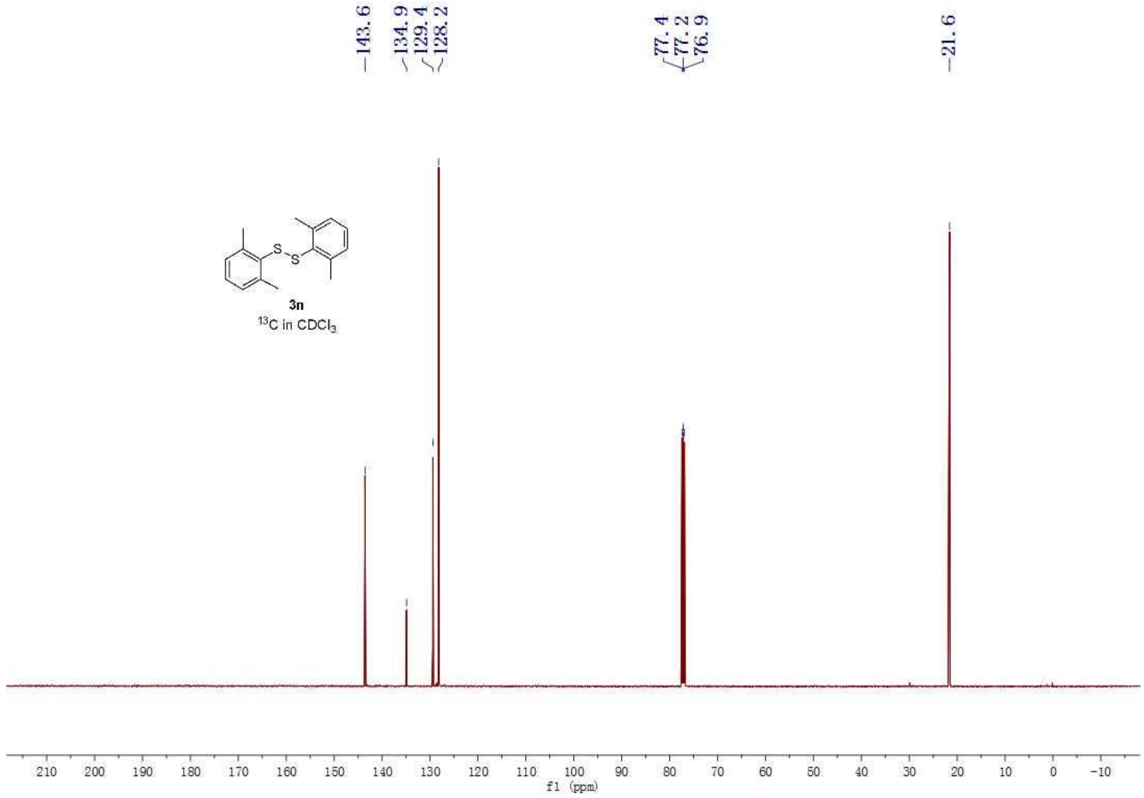
143.6
134.9
129.4
128.2
77.4
77.2
76.9
21.6
3n
13C in CDCl3
210 200 190 180 170 160 150 140 130 120 110 100 90 80 70 60 50 40 30 20 10 0 -10
f1 (ppm)

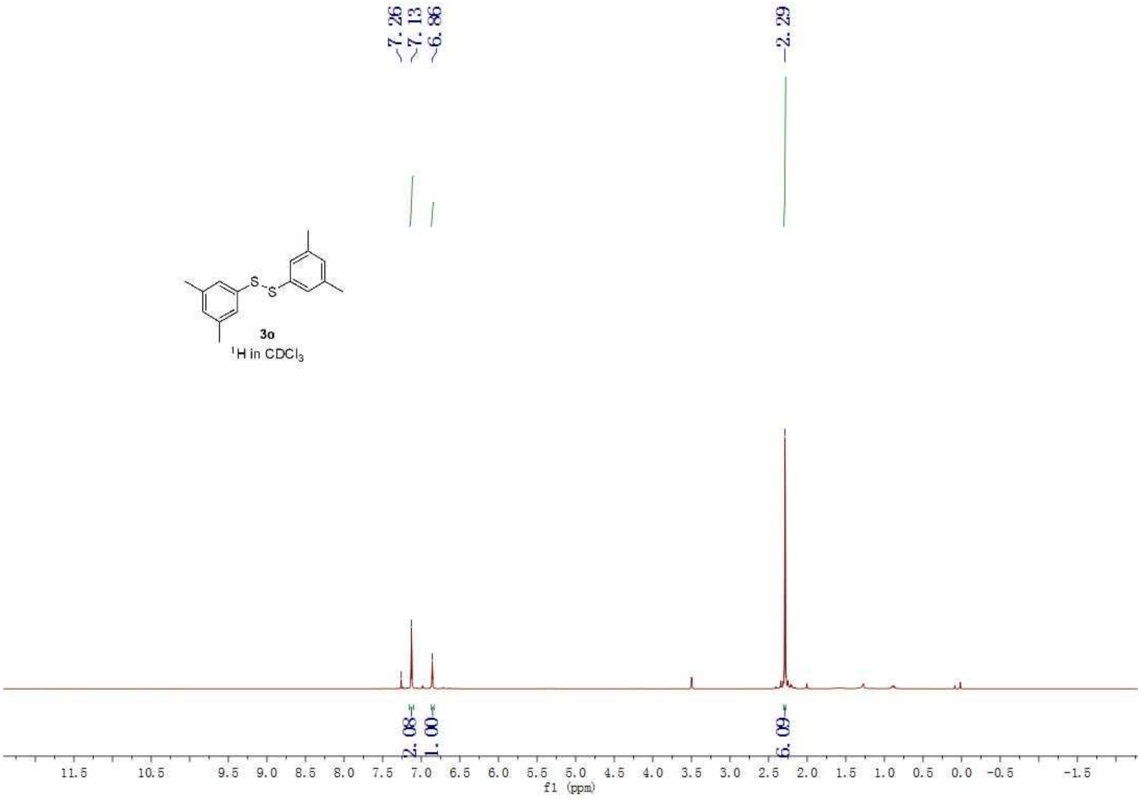
7.26
7.13
6.86
2.29
3o
^{1}H in $CDCl_3$
2.08
1.00
6.09
11.5 10.5 9.5 9.0 8.5 8.0 7.5 7.0 6.5 6.0 5.5 5.0 4.5 4.0 3.5 3.0 2.5 2.0 1.5 1.0 0.5 0.0 -0.5 -1.5
f1 (ppm)

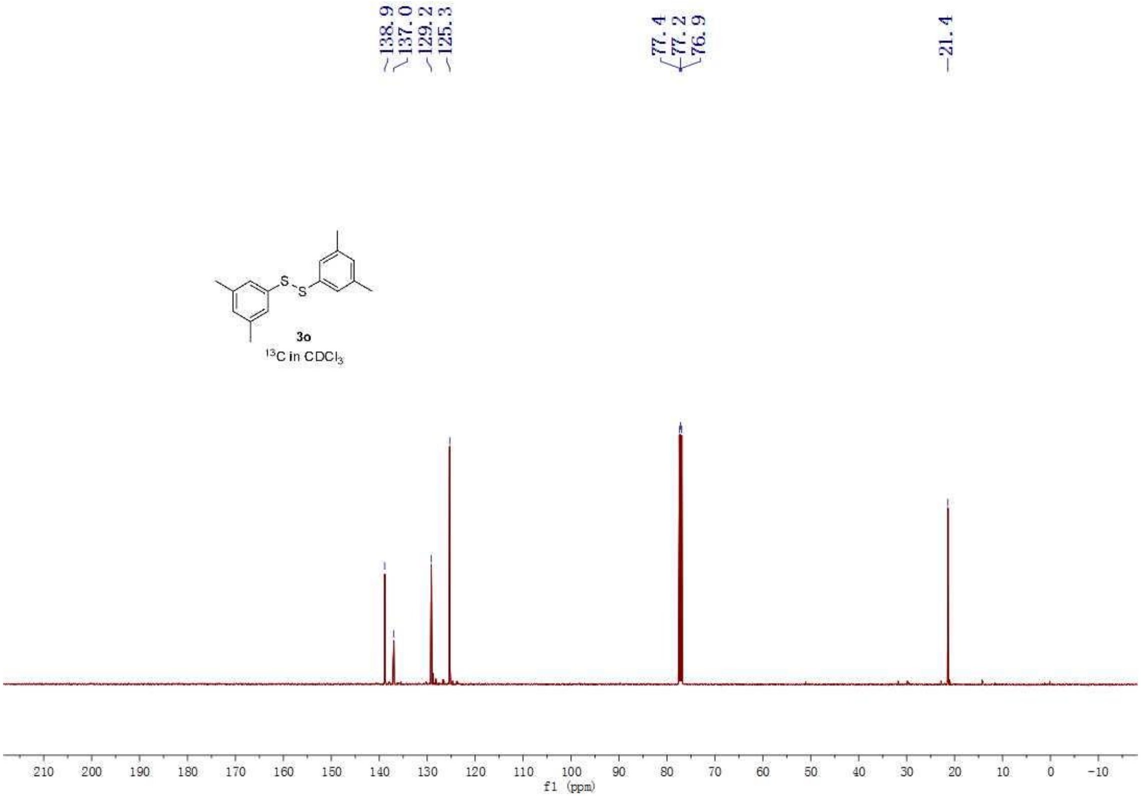
138.9
137.0
129.2
125.3
77.4
77.2
76.9
21.4
3o
^{13}C in $CDCl_3$
210 200 190 180 170 160 150 140 130 120 110 100 90 80 70 60 50 40 30 20 10 0 -10
f1 (ppm)

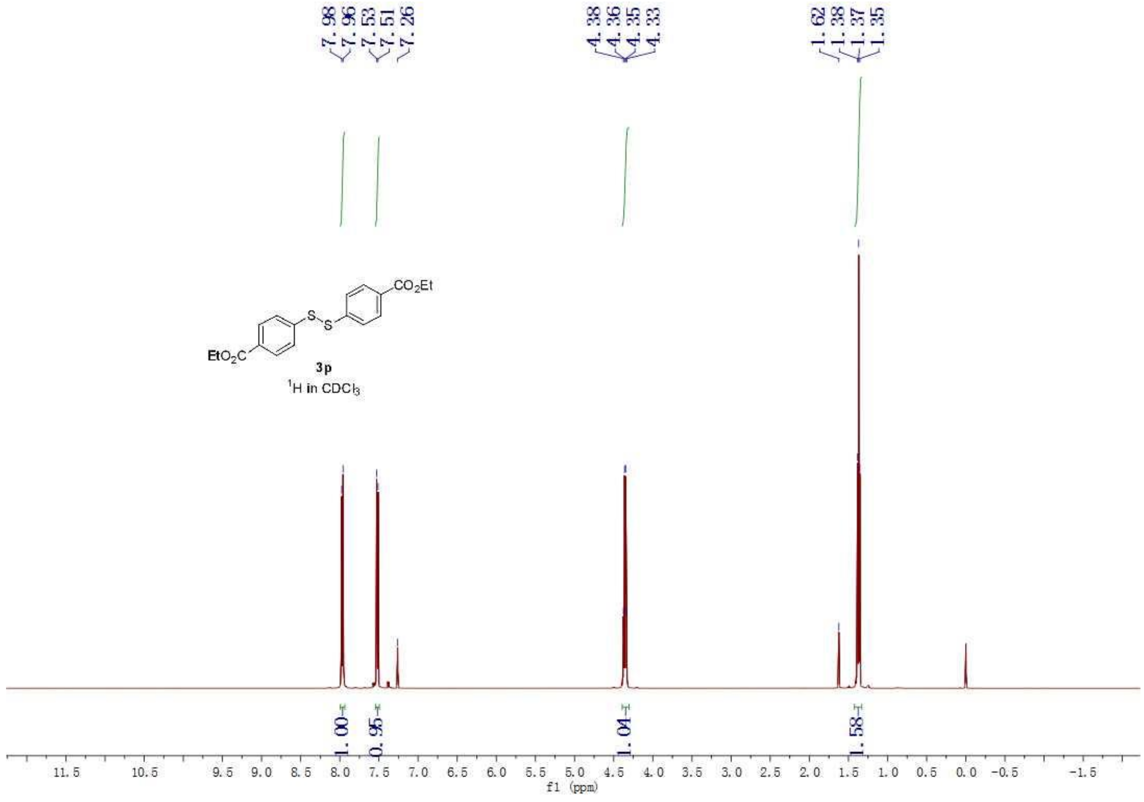
7.98
7.96
7.53
7.51
7.26
4.38
4.36
4.35
4.33
1.62
1.38
1.37
1.35
CO2Et
S
S
EtO2C
3p
1H in CDCl3
1.00
0.95
1.04
1.58
f1 (ppm)

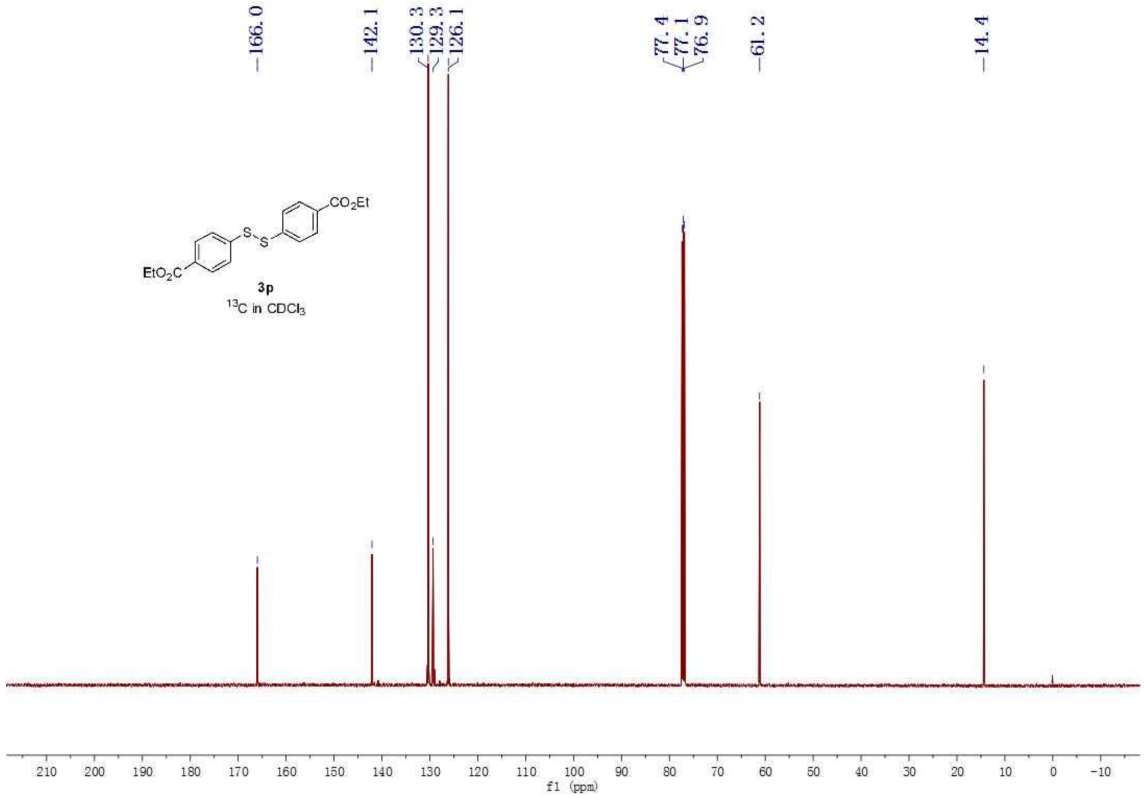
166.0
142.1
130.3
129.3
126.1
77.4
77.1
76.9
61.2
14.4
CO2Et
S
S
EtO2C
3p
13C in CDCl3
f1 (ppm)
